# New Staphylinidae (Coleoptera) records with new collection data from New Brunswick and an addition to the fauna of Quebec: Staphylininae

**DOI:** 10.3897/zookeys.186.2469

**Published:** 2012-04-26

**Authors:** Reginald P. Webster, Aleš Smetana, Jon D. Sweeney, Ian DeMerchant

**Affiliations:** 1 Natural Resources Canada, Canadian Forest Service - Atlantic Forestry Centre, 1350 Regent St., P.O. Box 4000, Fredericton, NB, Canada E3B 5P7; 2 Agriculture and Agri-Food Canada, Biodiversity, Central Experimental Farm, K. W. Neatby Bldg., Ottawa, ON K1A 0C6

**Keywords:** Staphylinidae, Staphylininae, new records, Canada, New Brunswick, United States, Maine

## Abstract

Forty-four species of Staphylininae are newly reported from New Brunswick, bringing the total number of species known from the province to 126. *Quedius criddlei* (Casey) is reported for the first time from Quebec. *Bisnius cephalotes* (Gravenhorst) is removed from the faunal list of New Brunswick due to a lack of supporting voucher specimens. Additional locality data are presented for seven species either recently recorded from the province or with few previous records and little habitat data. We provide the first documented records of *Atrecus americanus* (Casey), *Quedius erythrogaster* Mannerheim, *Quedius labradorensis labradorensis* Smetana, *Quedius plagiatus* (Mannerheim), and *Neobisnius terminalis* (LeConte) from New Brunswick. Collection and habitat data are presented and discussed for all species.

## Introduction

Intensive collecting of rove beetles (family Staphylinidae) in New Brunswick by the first author since 2003 has yielded many new provincial and national records. Additional records were obtained from by-catch samples during a study to develop a general attractant for detecting invasive species of Cerambycidae. These records are being published in a series of papers, each focusing on one or more subfamilies of Staphylinidae and other families of Coleoptera. This paper covers the subfamily Staphylininae.

This subfamily is fairly well known taxonomically in Canada and North America, thanks to various revisions. The genera *Erichsonius* and *Neobisnius* were revised by Frank (1975, 1981), respectively. The Xantholinini were revised by Smetana (1982, 1988), the Quediina by Smetana (1965, 1971a, 1971b, 1973, 1976, 1978, 1981, 1990), the Philonthina by Smetana (1995), and the Staphylinina by Brunke et al. (2011). As a result of these revisions, our knowledge of the distribution of these species in Canada was also greatly increased.

Adults of Staphylininae live in a wide variety of habitats. Many species live in and near wetlands, including river and brook margins, lakeshores, vernal pool and pond margins, bogs, marshes, sea beaches, and various synanthropic situations (Smetana 1971a, 1982, 1995). Depending on species, adults usually occur in leaf litter, sphagnum moss, and other kinds of debris in these wetland habitats, but some species are highly hygrophilic and occur in floating mats of moss or vegetation (Smetana 1971a, 1982, 1995). Other species are associated with decaying organic materials, including compost, decaying mushrooms, animal droppings, and carcasses. Some species are regular inhabitants of bird and mammal nests. *Philonthus janus* Smetana, *Philonthus couleensis* Hatch, and *Quedius campbelli* Smetana are commonly found in North American beaver (*Castor canadensis* Kuhl) lodges and muskrat (*Ondatra zibethicus* (L.)) nests, but these associations are not strict, and these species can also be found in adjacent wetland habitats (Smetana 1995). However, a few species such as *Heterothops marmotae* Smetana and *Bisnius lautus* (Casey) have much stronger associations (Smetana 1971b, 1995). These species live in underground burrows of rodents and are rarely found in other habitats. Most species are probably general predators (Smetana 1995), although additional research is required to elucidate the biology of most members of this subfamily.

Campbell and Davies (1991) reported 27 species of Staphylininae for New Brunswick. The number of species recorded from the province was increased to 77 species as a result of revisions by Smetana (1995), and new additions to the fauna by Klimaszewski et al. (2005), Majka and Smetana (2007), Majka and Klimaszewski (2008a, b), Majka et al. (2009), Majka and Brown (2010), and Smetana and Webster (2011). Majka et al. (2011) reported *Atrecus americanus* (Casey), *Quedius erythrogaster* Mannerheim, *Quedius labradorensis labradorensis* Smetana, *Quedius plagiatus* (Mannerheim), and *Neobisnius terminalis* (LeConte) as occurring in New Brunswick but did not provide any supporting references or data for the records. Here, we report an additional 44 species of Staphylininae for New Brunswick, bringing the total number of species known for the province to 126 (Table 1).

**Table 1.** Species of Staphylininae (Staphylinidae) recorded from New Brunswick, Canada.

Subfamily Staphylininae Latreille
Tribe Diochini Casey
*Diochus schaumi* Kraatz**
Tribe Othiini Thomson
*Atrecus americanus* (Casey)
*Atrecus macrocephalus* (Nordmann)
Tribe Xantholinini Erichson
*Gyrohypnus angustatus* Stephens
*Gyrohypnus campbelli* Smetana*
*Gyrohypnus fracticornis* (Müller)
*Hypnogyra gularis* (LeConte)**
*Leptacinus intermedius* Donisthorpe
*Neohypnus beckeri* Smetana**
*Neohypnus hamatus* (Say)
*Neohypnus obscurus* (Erichson)
*Nudobius cephalus* (Say)
*Oxybleptes kiteleyi* Smetana
*Phacophallus parumpunctatus* (Gyllenhal)**
*Stictolinus flavipes* (LeConte)
*Xantholinus linearis* (Olivier)
*Xestolinus abdominalis* Casey**
Tribe Staphylinini Latreille
Subtribe Quediina Kraatz
*Acylophorus (Amacylophorus) pratensis* LeConte**
*Acylophorus (Acylophorus) caseyi* Leng*
*Acylophorus (Acylophorus) pronus* Erichson
*Anaquedius vernix* (LeConte)
*Hemiquedius ferox* (LeConte)*
*Heterothops fusculus* LeConte
*Heterothops minor* Smetana*
*Heterothops pusio* LeConte**
*Quedius (Microsaurus) bicoloris* Smetana & Webster
*Quedius (Microsaurus) campbelli* Smetana**
*Quedius (Microsaurus) canadensis* (Casey)
*Quedius (Microsaurus) criddlei* (Casey)**
*Quedius (Microsaurus) erythrogaster* Mannerheim
*Quedius (Microsaurus) mesomelinus* (Marsham)
*Quedius (Microsaurus) peregrinus* (Gravenhorst)
*Quedius (Microsaurus) spelaeus* Horn
*Quedius (Quedius) curtipennis* Bernhauer
*Quedius (Quedius) labradorensis* Smetana
*Quedius (Quedionuchus) plagiatus* (Mannerheim)
*Quedius (Distichalius) capucinus* (Gravenhorst)*
*Quedius (Distichalius) cinctus* (Paykull)
*Quedius (Raphirus) frigidus* Smetana**
*Quedius (Raphirus) fulvicollis* (Stephens)**
*Quedius (Raphirus) rusticus* Smetana
*Quedius (Raphirus) simulator* Smetana**
Subtribe Staphylinina Latreille
*Creophilus maxillosus* (Linnaeus)
*Dinothenarus badipes* LeConte
*Dinothenarus capitatus* (Bland)
*Ontholestes cingulatus* (Gravenhorst)
*Platydracus cinnamopterus* Gravenhorst
*Platydracus cupripennis* (Melsheimer)
*Platydracus fossator* (Gravenhorst)
*Platydracus viridanus* (Horn)
*Staphylinus ornaticauda* LeConte*
*Tasgius ater* (Gravenhorst)
*Tasgius melanarius* (Heer)
Subtribe Xanthopygina Sharp
*Tympanophorus puncticollis* Erichson
Subtribe Philonthina Kirby
*Bisnius blandus* (Gravenhorst)
*Bisnius cephalicus* (Casey)**
*Bisnius palmi* (Smetana)*
*Bisnius quediinus* (Horn)**
*Bisnius siegwaldii* (Mannerhiem)
*Bisnius sordidus* (Gravenhorst)
*Cafius bistriatus* Erichson
*Erichsonius alumnus* Frank**
*Erichsonius inutilis* (Horn)**
*Erichsonius nanus* (Horn)
*Erichsonius parcus* (Horn)**
*Erichsonius patella* (Horn)*
*Erichsonius pusio* (Horn)**
*Erichsonius rosellus* Frank**
*Gabrius appendiculatus* Sharp
*Gabrius astutoides* (A. Strand)
*Gabrius brevipennis* (Horn)
*Gabrius fallaciosus* (Horn)**
*Gabrius microphthalmus* (Horn)
*Gabrius picipennis* (Mäklin)
*Gabrius ulpius* Smetana*
*Hesperus apicialis* Say**
*Laetulonthus laetulus* (Say)**
*Neobisnius jucundus* (Horn)**
*Neobisnius lathrobioides* (Baudi)**
*Neobisnius sobrinus* (Erichson)
*Neobisnius terminalis* (LeConte)
*Neobisnius villosulus* (Stephens)
*Philonthus aequalis* Horn**
*Philonthus boreas* Smetana**
*Philonthus caeruleipennis* (Mannerheim)
*Philonthus carbonarius* (Gravenhorst)
*Philonthus cognatus* Stephens
*Philonthus concinnus* (Gravenhorst)
*Philonthus couleensis* Hatch
*Philonthus cruentatus* (Gmelini)
*Philonthus debilis* (Gravenhorst)
*Philonthus discoideus* (Gravenhorst)
*Philonthus flavibasis* Casey**
*Philonthus flumineus* Casey
*Philonthus furvus* Nordman
*Philonthus fusiformis* Melsheimer
*Philonthus gracilior* Casey
*Philonthus hepaticus* Erichson
*Philonthus hudsonicus* Horn
*Philonthus janus* Smetana
*Philonthus jurgans* Tottenham
*Philonthus lindrothi* Smetana
*Philonthus lomatus* Erichson
*Philonthus monaeses* Smetana
*Philonthus neonatus* Smetana*
*Philonthus opacipennis* Notman
*Philonthus palliatus* (Gravenhorst)
*Philonthus politus* (Linnaeus)
*Philonthus pseudolus* Smetana**
*Philonthus quadricollis* Horn
*Philonthus rectangulus* Sharp
*Philonthus schwarzi* Horn
*Philonthus sericans* (Gravenhorst)
*Philonthus sericinus* Horn**
*Philonthus sessor* Smetana
*Philonthus sphagnorum* Smetana
*Philonthus spiniformis* Hatch
*Philonthus stictus* Hausen
*Philonthus subvirescens* Thomson
*Philonthus thoracicus* (Gravenhorst)
*Philonthus umbratilis* (Gravenhorst)
*Philonthus umbrinoides* Smetana*
*Philonthus validus* Casey
*Philonthus varians* (Paykull)
*Philonthus varro* Smetana
*Philonthus vulgatus* Casey*

**Notes:** *New to province; **New to Maritime provinces

## Methods and conventions

The following records are based in part on specimens collected as part of a general survey by the first author to document the Coleoptera fauna of New Brunswick. Additional provincial records were obtained from specimens contained in the collection at Natural Resources Canada’s Atlantic Forestry Centre in Fredericton, New Brunswick.

### Collection methods

Various collection methods were employed to collect the Staphylininae reported in this study. Details are outlined in Campbell (1973) and Webster et al. (2009, Appendix). Many specimens were also collected as by-catch in Lindgren 12-funnel traps (ConTech Inc., Delta, BC) baited with various attractants as part of a study to develop a general attractant for detecting invasive species of Cerambycidae. These traps mimic tree trunks and are often effective for sampling species of Coleoptera that live in microhabitats associated with standing trees (Lindgren 1983). Traps were suspended from rope tied between two trees separated by at least 2 m, with the collecting cup 30–50 cm above the ground. Collecting cups contained either a 50:50 mixture of propylene glycol and deionized water plus 0.5 ml/L of Kodak Photo-Flo 200 and 12.5 mg/L of Bitrex (in 2008) or a saturated salt solution with 1–2 drops of unscented dish detergent (in 2009 and 2010). Traps were baited with various lures reported as pheromones for longhorn species in the Cerambycinae subfamily (racemic 3-hydroxyhexan-2-one or racemic 3-hydroxyoctan-2-one) (Allison et al. 2004; Hanks et al. 2007) and/or high-release-rate ethanol lures (ConTech, Inc, Delta, BC), or were left unbaited. The effects of lure treatment on attraction (capture in Lindgren traps) of Staphylininae species and other by-catch species will be reported in separate papers. Samples were collected once weekly (2008 and 2009) or once every 2 weeks (2010), and specimens frozen until they were processed. A description of the habitat was recorded for all specimens collected during this survey. Locality and habitat data are presented exactly as on labels for each record. This information, as well as additional collecting notes, is summarized in the collection and habitat data section for each species.

### Specimen preparation

Examples of most species of Staphylininae were dissected to confirm their identity. The genital structures were dehydrated in absolute alcohol, mounted in Canada balsam on celluloid microslides, and pinned with the specimens from which they originated.

### Distribution

Distribution maps, created using ArcMap and ArcGIS, are presented for each species in New Brunswick. Every species is cited with current distribution in Canada and Alaska, using abbreviations for the state, provinces, and territories. New records for New Brunswick are indicated in bold under Distribution in Canada and Alaska. The following abbreviations are used in the text:

**Table:** 

**AK**	Alaska	**MB**	Manitoba
**YT**	Yukon Territory	**ON**	Ontario
**NT**	Northwest Territories	**QC**	Quebec
**NU**	Nunavut	**NB**	New Brunswick
**BC**	British Columbia	**PE**	Prince Edward Island
**AB**	Alberta	**NS**	Nova Scotia
**SK**	Saskatchewan	**NF & LB**	Newfoundland and Labrador*

*Newfoundland and Labrador are each treated separately under the current Distribution in Canada and Alaska.

Acronyms of collections referred to in this study are as follows:

**AFC** Atlantic Forestry Centre, Natural Resources Canada, Canadian Forest Service, Fredericton, New Brunswick, Canada

**CNC** Canadian National Collection of Insects, Arachnids and Nematodes, Agriculture and Agri-Food Canada, Ottawa, Ontario, Canada

**CCC **C. Chantal Collection, Varennes, Quebec, Canada

**NBM** New Brunswick Museum, Saint John, New Brunswick, Canada

**RWC** Reginald Webster Collection, Charters Settlement, New Brunswick, Canada

## Results

Forty-four species of Staphylininae are newly recorded from New Brunswick; *Bisnius cephalotes* (Gravenhorst) is removed from the faunal list of New Brunswick because of lack of a supporting voucher specimen. We provide the first documented records of *Atrecus americanus* (Casey), *Quedius erythrogaster* Mannerheim, *Quedius labradorensis labradorensis* Smetana, *Quedius plagiatus* (Mannerheim), and *Neobisnius terminalis* (LeConte) from New Brunswick. This brings the total number of species known from the province to 126 (Table 1). Thirty-three of these species are newly recorded for the Maritime provinces (New Brunswick, Nova Scotia, Prince Edward Island) of Canada. Additional locality data are presented for seven species either recently recorded from the province or having few previous records and little bionomic data. *Quedius criddlei* (Casey) is reported for the first time from Quebec.

### Species accounts

All records below are species newly recorded for New Brunswick, Canada, unless noted otherwise (additional records). Species followed by ** are newly recorded from the Maritime provinces of Canada.

The classification of the Staphylininae follows Bouchard et al. (2011).

### Subfamily Staphylininae, Latreille, 1802

#### Tribe Diochini Casey, 1906

##### Diochus schaumi

Kraatz, 1860**

http://species-id.net/wiki/Diochus_schaumi

Map 1

###### Material examined.

**New Brunswick, York Co.**, Upper Brockway, 45.5684°N, 67.0993°W, 23.IV.2006, R. P. Webster, forested black spruce bog, in sphagnum. (1, RWC)

###### Collection and habitat data.

The single New Brunswick specimen was collected from sphagnum in a forested black spruce (*Picea mariana* (Mill.) B.S.P.) bog. Elsewhere this species has been reported from forest litter and from wet moss and debris in wet habitats, such as swamps, marshes, bogs, and lake and stream margins (Smetana 1982). The single adult was collected by sifting sphagnum during late April.

###### Distribution in Canada and Alaska.

ON, QC, **NB** (Smetana 1982).

**Map 1.**
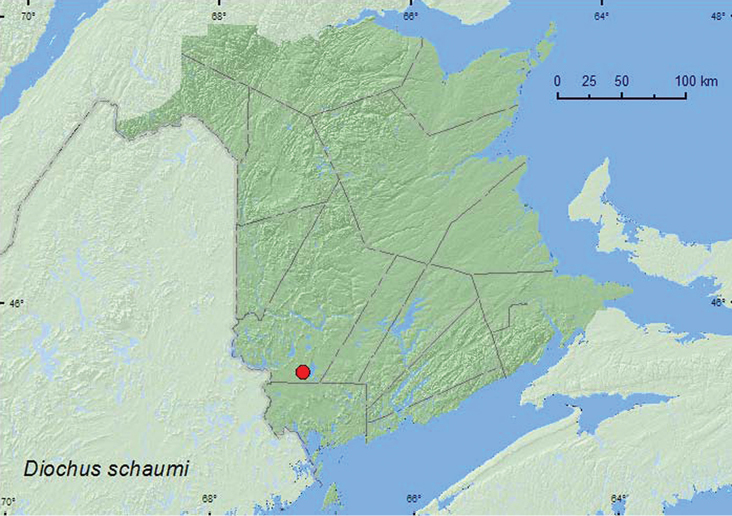
Collection localities in New Brunswick, Canada of *Diochus schaumi*.

#### Tribe Othiini Thomson, 1859

##### Atrecus americanus

(Casey, 1906)

http://species-id.net/wiki/Atrecus_americanus

Map 2

###### Material examined.

**Additional New Brunswick records, Albert Co.**, Caledonia Gorge P.N.A. (Protected Natural Area), at Turtle Creek, 45.8432°N, 64.8411°W, 5.VII.2011, A. Fairweather, old hardwood forest (sugar maple and beech), in rotten log near creek (1, NBM); Caledonia Gorge P.N.A. at Caledonia Creek, 45.7935°N, 64.7760°W, 1.VII.2011, R. P. Webster, shaded, rocky, cold, clear brook, splashing moss on rocks (1, NBM). **Carleton Co.**, Hovey Hill PNA, (Protected Natural Area), 46.1115°N, 67.7770°W, 19.VIII.2007, R. P. Webster, hardwood forest, under bark. (1, RWC); Jackson Falls, Bell Forest, 46.2208°N, 67.7211°W, 10.VII.2004, Vincent Webster & R. P. Webster, mixed forest, in bracket fungi (1, RWC); same locality and forest type but 46.2200°N, 67.7231°W, 13.VIII.2006, R. P. Webster, in decaying fleshy polypore fungi (1, RWC); same locality and forest type, 8–16.VI.2009, 16–21.VI.2009, 19–31.VII.2009, R. Webster & M.-A. Giguère, Lindgren funnel traps (3, AFC). **Queens Co.**, Cranberry Lake PNA, 46.1125°N, 65.6075°W, 21–27.V.2009, 21–28.VII.2009, 28.VII-6 VIII.2009, R. Webster & M.-A. Giguère, mature red oak forest, Lindgren funnel traps (3, AFC). **Restigouche Co.**, Berry Brook PNA, 47.8140°N, 66.7578°W, 26.V.2007, R .P. Webster, old-growth eastern white cedar swamp, in moss on hummock at base of cedar (2 ♂, RWC); Jacquet River Gorge PNA, 47.7491°N, 66.1114°W, 24.VI.2008, R. P. Webster, hardwood forest, under bark (1, NBM). **Sunbury Co.**, Acadia Research Forest, 45.9799°N, 66.3394°W, 14.V.2007, R. P. Webster, mature red spruce and red maple forest, sifting deep conifer litter at base of large red spruce (1, AFC); Acadia Research Forest, 45.9866°N, 66.3841°W, 2–9.VI.2009, 21–29.VII.2009, 29.VII-4.VIII.2009, R. Webster & M.-A. Giguère, mature (110-year-old) red spruce forest with scattered red maple and balsam fir, Lindgren funnel traps (3, AFC). **York Co.**, Charters Settlement, 45.8300°N, 66.7360°W, 1.V.2004, R. P. Webster, mixed forest, under bark of conifer log (1, RWC); same locality data and collector but 21.VI.2004, mixed forest, under bark (1, RWC); same locality and collector but 45.8286°N, 66.7211°W, 10.VII.2005, mature red spruce and cedar forest, in bracket fungi (1 ♂, RWC); Fredericton, Odell Park, 7.IX.2005, 45.9570°N, 66.6695°W, R. P. Webster, old-growth hemlock forest, on bracket fungi (1, RWC); 15 km W of Tracy off Rt. 645, 45.6848°N, 66.8821°W,1–8.VI.2009, R. Webster & M.-A. Giguère, old red pine forest, Lindgren funnel trap (1, AFC); 14 km WSW of Tracy, S of Rt. 645, 45.6741°N, 66.8661°W, 10–26.V.2010, R. Webster & C. MacKay, coll., old mixed forest with red and white spruce, red and white pine, balsam fir, eastern white cedar, red maple, and *Populus* sp., Lindgren funnel trap (1, AFC).

###### Collection and habitat data.

Smetana (1982) reported this species from under bark of dead trees with one record from *Fomitopsis pinicola* (Swartz: Fr.) Karst. In New Brunswick, this species was found in various forest types, including hardwood, mixed red spruce (*Picea rubens* Sarg.) and eastern white cedar (*Thuja occidentalis* L.), and old-growth hemlock (*Tsuga canadensis* (L.)). Adults were found under bark of both dead hardwood and conifer trees, in rotten logs, and in various species of polypore fungi (fleshy and bracket fungi). One adult was found in moss on a rock along a shaded brook. Adults were collected during May, June, July, August, and September.

###### Distribution in Canada and Alaska.

ON, QC, NB, NS (Smetana 1982). *Atrecus americanus* was listed as occurring in New Brunswick by Majka et al. (2011) without any supporting references or data. Here, we provide the first documented records from New Brunswick.

**Map 2.**
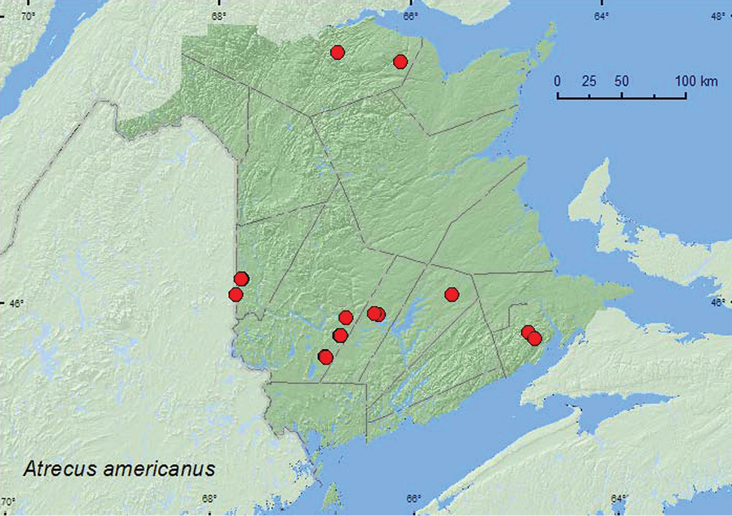
Collection localities in New Brunswick, Canada of *Atrecus americanus*.

#### Tribe Xantholinini Erichson, 1839

##### Gyrohypnus campbelli

Smetana, 1982

http://species-id.net/wiki/Gyrohypnus_campbelli

Map 3

###### Material examined.

**New Brunswick, Restigouche Co.**, Jacquet River Gorge P.N.A, 47.7146°N, 66.1644°W, 24.VI.2008, R. P. Webster, alder swamp adjacent to slow flowing brook, in leaves on muddy soil (1 ♂, NBM). **York Co.**, Charters Settlement, 45.8428°N, 66.7279°W, 19.IV.2005, 28.IV.2004, R. P. Webster, mixed forest, in litter near small sedge marsh (2, RWC); same locality but 45.8395°N, 66.7391°W, 23.IV.2004, 29.IV.2004, 9.V.2005, R. P. Webster, mixed forest near small shaded brook, in forest litter (2 ♂, 3 sex undetermined, NBM, RWC); same locality but 45.8341°N, 66.7445°W, 22.IV.2005, R. P. Webster, mature red spruce and eastern white cedar forest, margin vernal pond in leaf litter (2, RWC); New Maryland, off Hwy 2, E of Baker Brook, 45.8760°N, 66.6252°W, 6.IV.2005, R. P. Webster, old growth eastern cedar swamp in moss and litter on hummock (1, RWC); Rt. 645 at Beaver Brook, 45.6860°N, 66.8668°W, R. P. Webster, *Carex* marsh, in litter at base of dead red maple (1 ♂, RWC); near Mazerolle Settlement, NE of exit 271, off Hwy 2, 45.8776°N, 66.8254°W, 8.VI.2008, R. P. Webster, alder swamp with poplar, in leaf litter and moss near vernal pool (1, NBM).

###### Collection and habitat data.

In New Brunswick, adults were usually found in moss, leaf, and grass litter near various kinds of wet habitats. These included *Carex* marshes, shaded brook margins, a vernal pond in a mature red spruce and eastern white cedar forest, and on hummocks in an old-growth eastern white cedar swamp. Elsewhere, this species has been found in similar habitats, including a series collected from a beaver lodge (Smetana 1982). Adults were collected during April, May, and June.

###### Distribution in Canada and Alaska.

MB, ON, QC, **NB**, NS (Smetana 1982; Bishop et al. 2009).

**Map 3.**
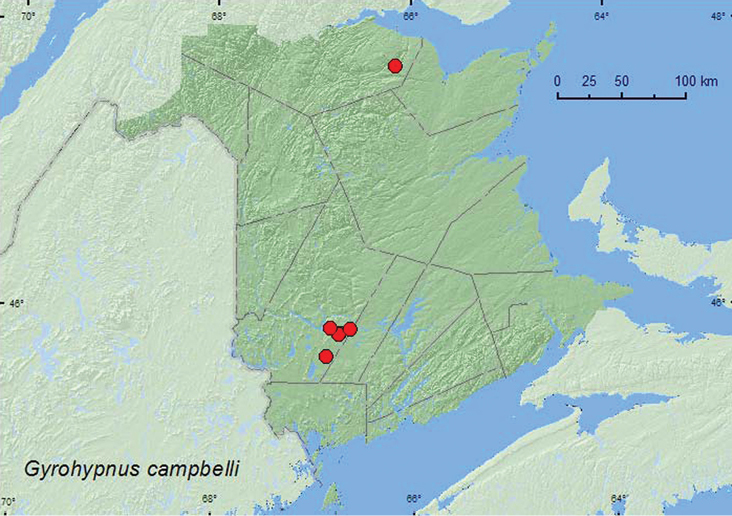
Collection localities in New Brunswick, Canada of *Gyrohypnus campbelli*.

##### Hypnogyra gularis

(LeConte, 1880)**

http://species-id.net/wiki/Hypnogyra_gularis

Map 4

###### Material examined.

**New Brunswick, Carleton Co.**, Jackson Falls, Bell Forest, 46.2200°N, 67.7231°W, 18.IX.2006, R. P. Webster, mature hardwood forest, under bark of large dead basswood covered with polypore fungi (1, RWC); same locality and habitat, 20–26.V.2009, R. Webster & M.-A. Giguère, Lindgren funnel trap (1, RWC). **Queens Co.**, Rees, near Grand Lake, 46.0016°N, 65.9466°W, 29.V.2007, S. Makepeace & R. P. Webster, nest box contents of barred owl (1, RWC); Cranberry Lake P.N.A., 46.1125°N, 65.6075°W, 5–12 V.2009, R. Webster & M.-A. Giguère, mature red oak forest, Lindgren funnel trap (1, RWC).

###### Collection and habitat data.

Little is known about the habitat assciations of members of this genus, especially in North America (Smetana 1982). Species from the Palaearctic have been collected from hollows of trees and stumps and in litter at bases of dead trees; some species occur regularly in bird nests in hollow trees and are often observed among ants (Smetana 1982). In New Brunswick, one specimen was collected from the nest contents of a barred owl (*Strix varia* Barton), one from under the bark of a large, dead, fungus-covered basswood (*Tilia americana* L.) log, and another from a Lindgren funnel trap deployed in mature (old) red oak (*Quercus rubra* L.) forest. One specimen was collected in association with an ant, *Formica subsericea* Say, in Iowa City, Iowa (Smetana 1982). Adults were collected during May and September.

###### Distribution in Canada and Alaska.

ON, **NB** (Smetana 1988).

**Map 4.**
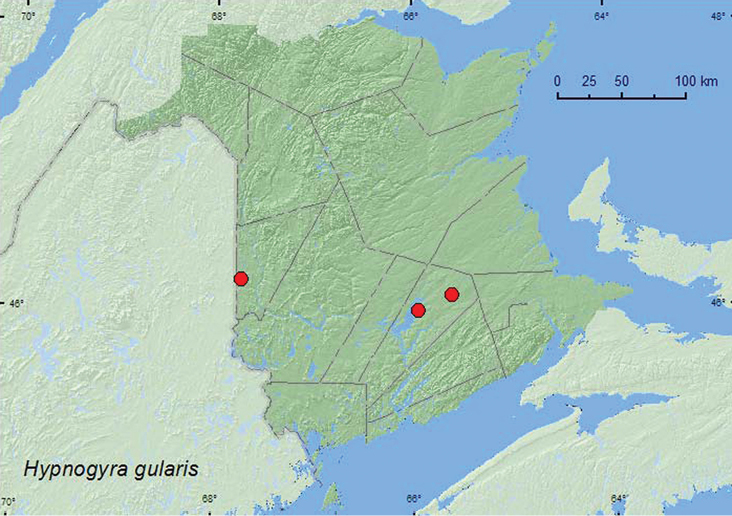
Collection localities in New Brunswick, Canada of *Hypnogyra gularis*.

##### Neohypnus beckeri

Smetana, 1982**

http://species-id.net/wiki/Neohypnus_beckeri

Map 5

###### Material examined.

**New Brunswick, Carleton Co.**, Jackson Falls, Bell Forest, 46.2200°N, 67.7231°W, 23–28.IV.2009, 20–26.V.2009, R. Webster & M.-A. Giguère, mature hardwood forest, Lindgren funnel traps (3, AFC). **Charlotte Co.**, 10 km NW of New River Beach, 45.2110°N, 66.6170°W, 30.IV-17.V.2010, R. Webster & V. Webster, coll., old growth eastern white cedar forest, Lindgren funnel trap (1, AFC). **Queens Co.**, Cranberry Lake P.N.A., 46.1125°N, 65.6075°W, 12–21.V.2009, 5–11.VI.2009, R. Webster & M.-A. Giguère, mature red oak forest, Lindgren funnel traps (2, AFC). **Sunbury Co.**, Sunpoke Lake, 45.7665°N, 66.5545°W, 15.V.2004, R. P. Webster, red oak and maple forest, under coyote scat and in leaf litter (2, RWC); Acadia Research Forest, 46.0188°N, 66.3765°W, 14.V.2008, 18.VI.2009, R. P. Webster, mature red spruce and red maple forest, sifting leaf litter (2, AFC); Acadia Research Forest, 45.9866°N, 66.3841°W, 13–19.V.2009, 19–25.V.2009, 25.V-2.VI.2009, 2–9.VI.2009, R. Webster & M.-A. Giguère, mature (100-year-old) red spruce forest with scattered red maple and balsam fir, Lindgren funnel traps (6, AFC); *ca*. 5 km SE of Geary, 45.7057°N, 66.4432°W, 22.V.2009, S. Makepeace & R. Webster, in leaf litter with scat & bones under tree with active great horned owl nest (1, NBM). **York Co.**, Charters Settlement, 45.8304°N, 66.7351°W, 18.IV.2004, 6.V.2004, R. P. Webster, mixed forest, under moist cardboard covering old chicken bones (2, RWC); same locality but 45.8395°N, 66.7391°W, 5.V.2004, 12.V.2004, 9.V.2004, R. P. Webster, mixed forest, in compost (decaying vegetables) (3, RWC); same locality but 45.8286°N, 66.7365°W, 7.VI.2005, R. P. Webster, mature red spruce and eastern white cedar forest, in leaf litter (2 , RWC); Canterbury, “Browns Mountain Fen Complex”, 45.8937°N, 67.6564°W, 8.VI.2004, D. Sabine & R. Webster, black spruce bog with eastern white cedar, in moist sphagnum on bog margin (1, RWC); 14 km WSW of Tracy, S of Rt. 645, 45.6741°N, 66.8661°W, 26.IV-10.V.2010, R. Webster & C. MacKay, coll., old mixed forest with red and white spruce, red and white pine, balsam fir, eastern white cedar, red maple, and *Populus* sp., Lindgren funnel traps (2, AFC).

###### Collection and habitat data.

In New Brunswick, adults were collected from under coyote scat, under cardboard covering old chicken bones, in compost, in leaf litter in hardwood and mixed forests, and in moist sphagnum on the margin of a black spruce and eastern white cedar bog/fen. Smetana (1982) reported most specimens from leaf litter in deciduous forests, although a few were reported from a human dung trap and from under a dead beaver. Adults were collected during April, May, and June.

###### Distribution in Canada and Alaska.

ON, QC, **NB** (Smetana 1982).

**Map 5.**
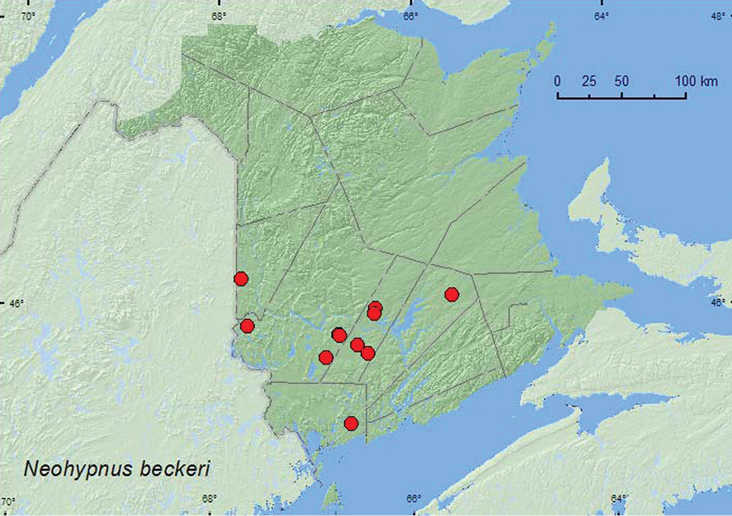
Collection localities in New Brunswick, Canada of *Neohypnus beckeri*.

##### Phacophallus parumpunctatus

(Gyllenhal, 1827)**

http://species-id.net/wiki/Phacophallus_parumpunctatus

Map 6

###### Material examined.

**New Brunswick, York Co.**, Charters Settlement, 45.8395°N, 66.7391°W, 16.X.2004, R. P. Webster, mixed forest, in compost (decaying vegetables) (1, RWC).

###### Collection and habitat data.

In Europe, this species is synanthropic, occurring in compost, manure, and other decaying organic material (Smetana 1982). In North America, specimens of this adventive species were collected from decaying grass clippings and from a pile of moldy wood chips and damp decaying vegetation (Smetana 1982). The single specimen from New Brunswick was collected from decaying vegetables (compost) in October.

###### Distribution in Canada and Alaska.

ON, QC, **NB** (Smetana 1982).

**Map 6.**
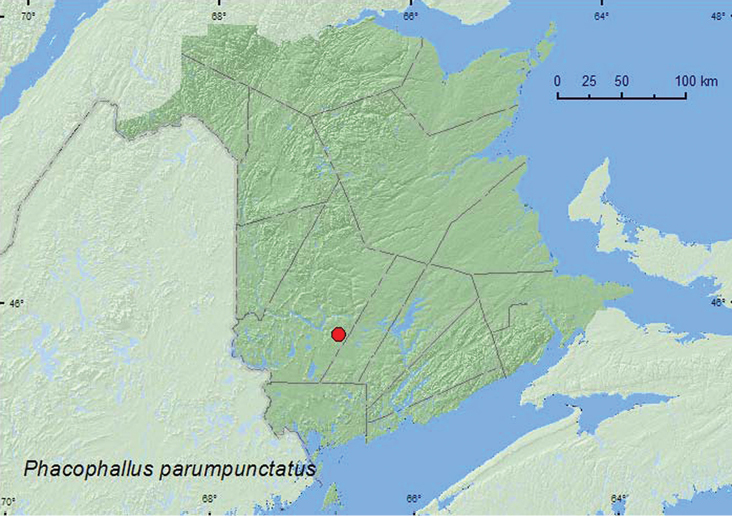
Collection localities in New Brunswick, Canada of *Phacophallus parumpunctatus*.

##### Xestolinus abdominalis

Casey, 1906**

http://species-id.net/wiki/Xestolinus_abdominalis

Map 7

###### Material examined.

**New Brunswick, Sunbury Co.**, Acadia Research Forest, 46.0188°N, 66.3765°W, 14.V.2007, R. P. Webster, mixed forest, in flight, collected with net (1 sex undetermined, RWC). **York Co.**, Charters Settlement, 45.8267°N, 66.7343°W, 30.IV.2005, R. P. Webster, *Carex* marsh, in sphagnum hummock (1 sex undetermined, RWC).

###### Collection and habitat data.

Little is known about the habitat associations of this species. One of the New Brunswick specimens was collected from a sphagnum hummock in a *Carex* marsh, another was collected while it was flying in a mixed forest near a *Carex* marsh. Adults were collected in late April and May.

###### Distribution in Canada and Alaska.

SK, MB, ON, QC, **NB** (Smetana 1982).

**Map 7.**
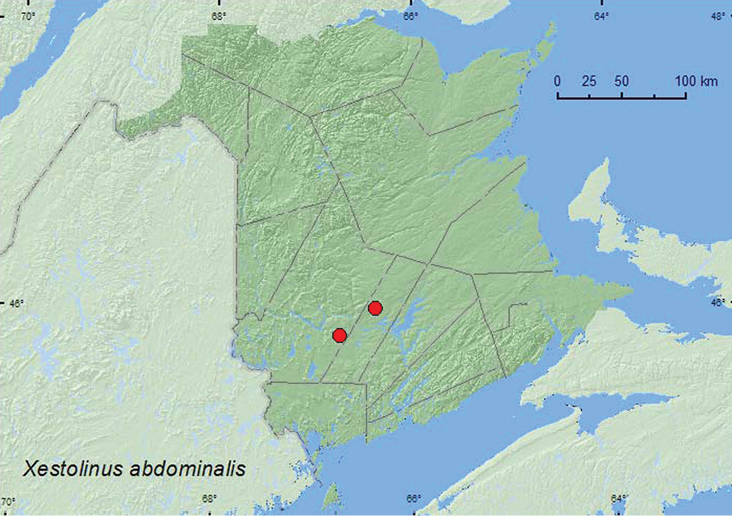
Collection localities in New Brunswick, Canada of *Xestolinus abdominalis*.

#### Tribe Staphylinini Latreille, 1802

##### Subtribe Quediina Kraatz, 1857

###### Acylophorus (Amacylophorus) pratensis

LeConte, 1863**

http://species-id.net/wiki/Acylophorus_pratensis

Map 8

####### Material examined.

**New Brunswick, Carleton Co.**, Jackson Falls, Bell Forest, 46.2150°N, 67.7190°W, 12.VI.2008, R. P. Webster, river margin, treading vegetation in seepage area (1, NBM). **Charlotte Co.**, 3.5 km NW of Pomeroy Ridge, 45.3087°N, 67.4362°W, 16.VI.2008, R. P. Webster, red maple swamp, in leaves and moss near small vernal pool (1, NBM). **Northumberland Co.,** Goodfellow Brook P.N.A., 46.8943°N, 65.3796°W, 23.V.2007, R. P. Webster, old growth eastern white cedar swamp, in grass litter and moss on hummocks near pool (1 ♂, 1 sex undetermined, NBM, RWC). **Restigouche Co.**, Jacquet River Gorge P.N.A. at Jacquet River, 47.7765°N, 66.1277°W, 13.VIII.2010, R. P. Webster, in moss on rocks in middle of river, splashing rocks (1, NBM). **York Co.**, Charters Settlement, 45.8395°N, 66.7391°W, 23.IV.2004, 3.VI.2004, 9.V.2005, 6.X.2005, R. P. Webster, mixed forest, in litter and moss near margin of small shaded brook (5, RWC); same locality and collector but 45.8283°N, 66.7350°W, 8.V.2004, sedge marsh, in sphagnum hummock (1, RWC); same locality and collector but 45.8428°N, 66.7279°W, 23.VI.2004, 20.IV.2005, mixed forest, (shaded) margin of small sedge marsh in moist sphagnum (2, RWC); 8.4 km W of Tracy off Rt. 645, 45.6821°N, 66.7894°W, 14.V.2008, R. P. Webster, wet alder swamp, in grass hummock (1, RWC).

####### Collection and habitat data.

In New Brunswick, most adults were collected in red maple (*Acer rubrum* L.) swamps, alder (*Alnus* sp.) swamps, and eastern white cedar swamps, usually along vernal pool and brook margins, and in *Carex* marshes. One adult was collected from a seepage area along a river margin. Adults were sifted from moss and various kinds of grass and leaf litter. Elsewhere, specimens were collected from dead swamp grass and moss (Smetana 1971a), leaf litter in a cedar bog, and a pitfall trap in a bog (Smetana 1981). Adults in New Brunswick were collected in April, May, and June.

####### Distribution in Canada and Alaska.

ON,QC, **NB,** NF (Smetana 1971a, 1973, 1981).

**Map 8.**
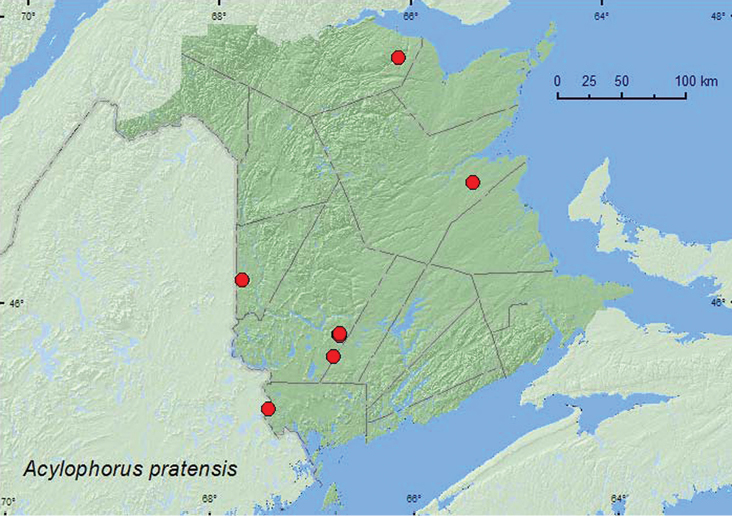
Collection localities in New Brunswick, Canada of *Acylophorus pratensis*.

###### Acylophorus (Amacylophorus) caseyi

Leng, 1920

http://species-id.net/wiki/Acylophorus_caseyi

Map 9

####### Material examined.

**New Brunswick, Charlotte Co.**, Rt. 3 at Deadwater Brook, 45.4744°N, 67.1225°W, 3.VI.2005, R. P. Webster, forested black spruce bog, marshy stream margin, treading (1, RWC); near New River, 45.1616°N, 66.6649°W, 7.VII.2006, R. P. Webster, treading sedge marsh (1, NBM); near Clark Ridge, 45.3155°N, 67.4406°W, 27.V.2007, R. P. Webster, beaver pond, treading vegetation (1, NBM). **Queens Co.**, Upper Gagetown, bog adjacent to Hwy 2, 45.8316°N, 66.2346°W, R. P. Webster, tamarack bog, in sphagnum hummock and litter at bog margin (1 ♂, 2 sex undetermined, NBM, RWC). **York Co.**, Charters Settlement, 45.8267°N, 66.7343°W, 8.V.2004, 16.IV.2005, 30.IV.2005, 14.V.2005, R. P. Webster, *Carex* marsh in sphagnum hummocks (5, RWC).

####### Additional Maine Record.

**Penobscot Co.**, T6 R8 WELS, Marble Fen, 46.1245°N, 68.6983°W, 13.VI.2003, P. deMaynadier and R. Webster, open wet tamarack bog, in moist sphagnum (treading) (1, NBM).

####### Collection and habitat data.

Smetana (1971a, 1976) reported this species from swampy and marshy areas, and along lake and bog margins. Adults occurred in wet moss, wet sphagnum, floating sphagnum mats, leaves and debris, and other floating vegetation. In Nova Scotia, adults were collected in a eutrophic, *Typha latifolia* L. marsh (Smetana 1965). In New Brunswick, this species was found in marsh vegetation or saturated sphagnum hummocks along a marshy stream margin near a forested black spruce bog, a tamarack (*Larix laricina* (Du Roi) Koch) bog, a beaver pond, and in *Carex* marshes. Most adults were collected by treading vegetation into water. Adults were collected in April, May, June, and July.

####### Distribution in Canada and Alaska.

ON,QC, **NB**, NS (Smetana 1971a, 1973, 1976). Smetana (1990) reported *Acylophorus caseyi* from western Maine near the border with New Hampshire (Wilsons Mills Bog, Oxford Co.). The above record from Maine represents a significant eastern range extension in the state. It is apparent from the above records that *Acylophorus caseyi* probably has a more continuous distribution in the Northeast and the Maritime provinces as a whole than was suggested by the collection records reported in Majka et al. (2009). These distributional gaps likely reflect incomplete collecting effort in the appropriate wetland habitats. One must use treading to collect this species from the wetland habitats that this species usually frequents.

**Map 9.**
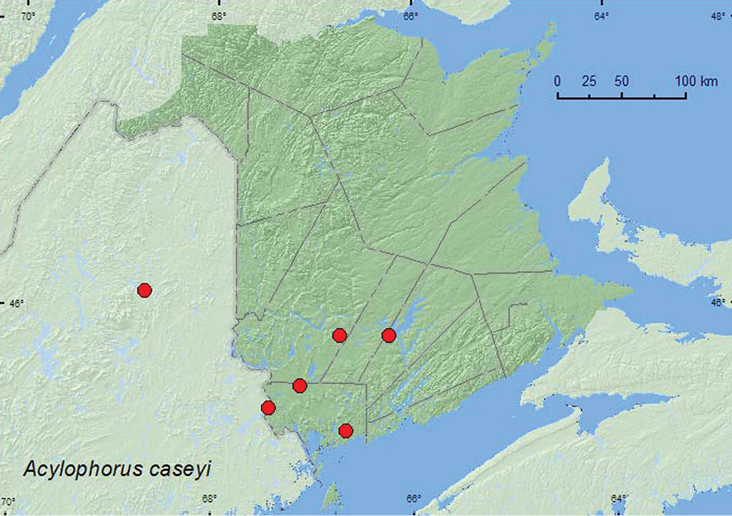
Collection localities in New Brunswick, Canada and Maine, United States of America of *Acylophorus caseyi*.

###### Hemiquedius ferox

(LeConte, 1878)

http://species-id.net/wiki/Hemiquedius_ferox

Map 10

####### Material examined.

**New Brunswick, Carleton Co.**, Jackson Falls, 46.2257°N, 67.7420°W, 22.V.2010, R. P. Webster, river margin, in gravel near water on gravel bar (1, NBM). **Charlotte Co.**, 3.0 km NW of Pomeroy Ridge, 45.3059°N, 67.4343°W, 5.VI.2008, R. P. Webster, alder swamp, in moss hummocks with grasses (2, NBM, RWC); near New River, 45.2118°N, 66.6179°W, 7.VII.2008, R. P. Webster, mixed forest, margin of small pond, treading *Carex* hummock (1, RWC). **Queens Co.**, Grand Lake Meadows P.N.A., 45.8227°N, 66.1209°W, 24.VIII-3.IX.2010, C. Hughes & K. Burgess, old silver maple forest with green ash and seasonally flooded marsh, Lindgren funnel trap (1, AFC). **Sunbury Co.**, Maugerville, Portobello Creek N.W.A., 45.8992°N, 66.4248°W, 27.V.2004, 5.VI.2004, R. P. Webster, silver maple swamp, margin of small vernal pond in leaf litter (2, RWC); near Sunpoke Lake, 45.7662°N, 66.5526°W, 20.VI.2007, R. P. Webster, seasonally flooded marsh, treading (1 sex undetermined, RWC). **York Co.**, Fredericton, University of New Brunswick Woodlot, 45.9391°N, 66.6747°W, 17.VIII.2009, R. Webster, D. McAlpine, & G. Forbes, within wall of a beaver (*Castor canadensis*) lodge (4 ♀, RWC).

####### Collection and habitat data.

*Hemiquedius ferox*was reported by Smetana (1971a) from various wet habitats, such as swamps, lake margins, and marshes. Adults were found in wet moss and leaves and in beaver lodges in late fall. Adults were found in similar habitats in New Brunswick, including an alder swamp, silver maple (*Acer saccharinum* L.) swamps, pond margins, a seasonally flooded marsh, a gravel bar on river, and a beaver lodge. Adults occurred in moss and *Carex* hummocks, in leaf litter along vernal pond margins and within the wall of a beaver lodge. Adults were collected by sifting litter and moss or treading vegetation into water. One individual was collected in a Lindgren funnel trap. Adults were captured during May, June, July, August, and September.

####### Distribution in Canada and Alaska.

ON, QC, **NB**, NS (Smetana 1971a). It is apparent from the above records that *Hemiquedius ferox* is more widely distributed in the Maritime provinces than was suggested by the collection records reported in Majka et al. (2009).

**Map 10.**
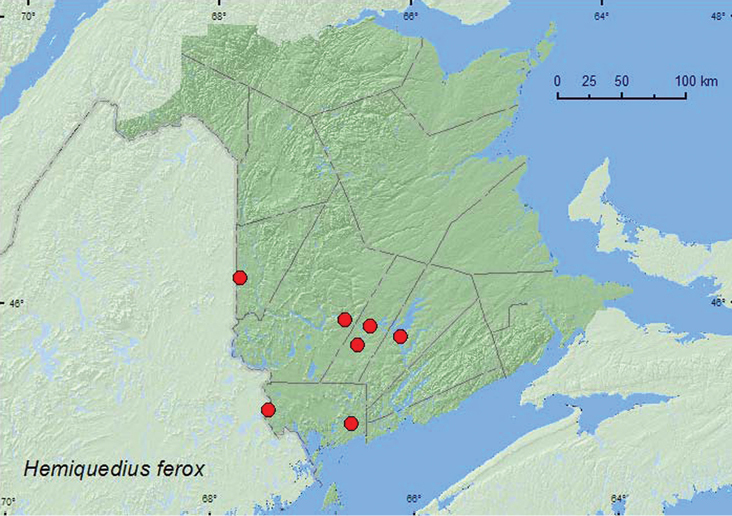
Collection localities in New Brunswick, Canada of *Hemiquedius ferox*.

###### Heterothops minor

Smetana, 1971

http://species-id.net/wiki/Heterothops_minor

Map 11

####### Material examined.

**New Brunswick, York Co.**, Upper Brockway, 45.5684°N, 67.0993°W, 23.IV.2006, R. P. Webster, forested black spruce bog, in sphagnum (1, RWC).

####### Collection and habitat data.

Little is known about the habitat associations of this species.Smetana (1976) reported specimens from under driftwood on a muddy river bank and from a pitfall trap on the margin of swampy pool in the Northwest Territories. The single adult from New Brunswick was sifted from sphagnum in a forested black spruce bog in late April.

####### Distribution in Canada and Alaska.

NT**,** BC, AB, MB, ON, QC, **NB**, NS, NF (Smetana 1971a, 1973, 1976, 1981).

**Map 11.**
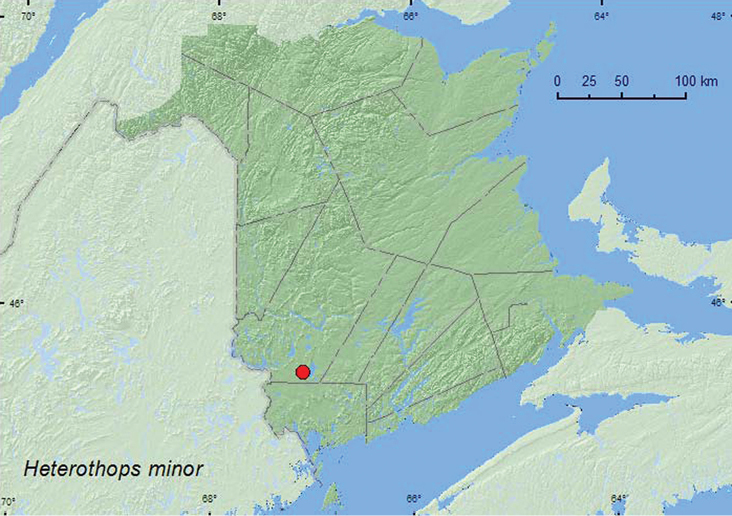
Collection localities in New Brunswick, Canada of *Heterothops minor*.

###### Heterothops pusio

LeConte, 1863**

http://species-id.net/wiki/Heterothops_pusio

Map 12

####### Material examined.

**New Brunswick, Carleton Co.**, Jackson Falls, Bell Forest, 46.2152°N, 67.7190°W, 1.VI.2005, M.-A. Giguère & R. Webster, river margin, flying adults collected with aerial net between 16:00 and 18:00 h (1 ♂, 1 ♀, RWC). **Restigouche Co.**, Jacquet River Gorge P.N.A., 47.8197°N, 66.0835°W, 23.VI.2008, R. P. Webster, river margin, among cobblestones (1, NBM). **York Co.**, Charters Settlement, 45.8395°N, 66.7391°W, 10.VI.2006, R. P. Webster, mixed forest, m.v. light (3, RWC); same locality data, collector, and forest type, 26.V.2008, compost (decaying vegetables) (1, RWC).

####### Collection and habitat data.

Smetana (1971a) reported this species from compost and grass piles, leaf and ground litter, and from old deserted beaver lodges. In New Brunswick, adults were sifted from compost and among cobblestones along a river margin. Other individuals were collected at a mercury-vapor light and during a late afternoon aerial flight. Adults were collected in May and June.

####### Distribution in Canada and Alaska.

BC, ON, QC, **NB** (Smetana 1971a, 1973, 1981).

**Map 12.**
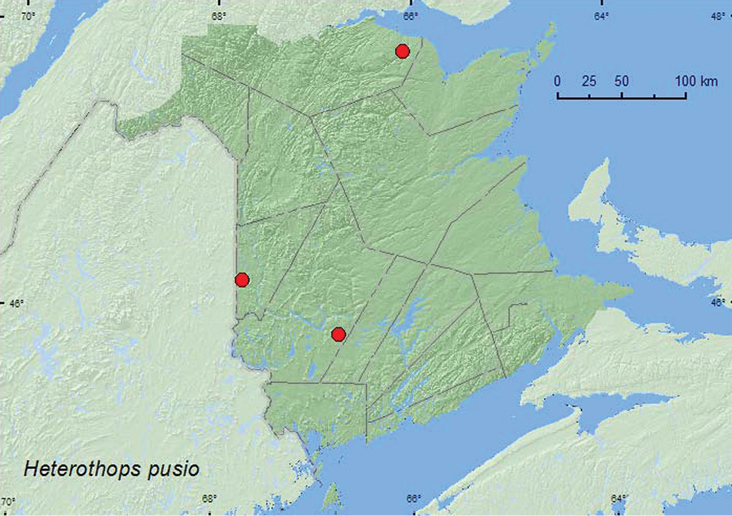
Collection localities in New Brunswick, Canada of *Heterothops pusio*.

###### Quedius (Microsaurus) campbelli

Smetana,1971**

http://species-id.net/wiki/Quedius_campbelli

Map 13

####### Material examined.

**New Brunswick, Restigouche, Co.**, Dionne Brook P.N.A., 47.9064°N, 68.3441°W, 31.V-15.VI.2011, M. Roy & V. Webster, old-growth white spruce and balsam fir forest, Lindgren funnel trap (1 ♀, RWC). **York Co.**, Fredericton, University of New Brunswick Woodlot, 45.9391°N, 66.6747°W, 17.VIII.2009, R. P. Webster, D. McAlpine, & G. Forbes, in beaver (*Castor canadensis*) lodge, within wall of lodge (2 ♂, NBM, RWC).

####### Collection and habitat data.

Smetana (1971a, 1976) reported specimens from near or within (in walls) muskrat nests and beaver lodges. Two of the New Brunswick specimens were collected from within the wall of a beaver lodge in August. One was captured during June in a Lindgren funnel trap deployed in an old balsam fir (*Abies balsamea* (L.) Mill.) and white spruce (*Picea glauca* (Moench) Voss) forest.

####### Distribution in Canada and Alaska.

ON**,** QC, **NB** (Smetana 1971a, 1976).

**Map 13.**
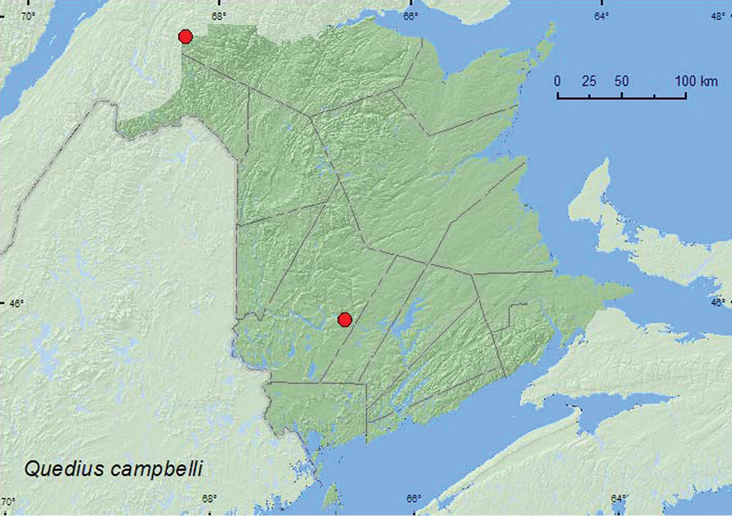
Collection localities in New Brunswick, Canada of *Quedius campbelli*.

###### Quedius (Microsaurus) canadensis

(Casey, 1915)

http://species-id.net/wiki/Quedius_canadensis

Map 14

####### Material examined.

**Additional New Brunswick records. Carleton Co.**, Jackson Falls, Bell Forest, 46.2200°N, 67.7231°W, 18.IX.2006, R. P. Webster, mature hardwood forest, under bark of large, dead, standing basswood covered with bracket fungi (1 ♂, RWC); same locality, 4–12.VI.2008, 12–19.VI.2008, 5–12.VII.2008, 23–28.IV.2009, 9–14.V.2009, 14–20.V.2009, 9–16.VI.2009, R. Webster & M.-A. Giguère, mature hardwood forest, Lindgren funnel traps (2 ♂, 3 ♀, 6 sex undetermined, AFC, NBM, RWC). **Charlotte Co.**, 10 km NW of New River Beach, 45.2110°N, 66.6170°W, 30.IV-17.V.2010, R. P. Webster & V. Webster, coll., old-growth eastern white cedar forest, Lindgren funnel trap (1, AFC). **Queens Co.**, Cranberry Lake P.N.A., 46.1125°N, 65.6075°W, 24.IV-5 V.2009, R. Webster & M.-A. Giguère, mature red oak forest, Lindgren funnel traps (5, AFC, NBM). **Restigouche Co.**, Jacquet River Gorge P.N.A., 47.8160°N, 66.0083°W, 14.VIII.2010, R. P. Webster, old eastern white cedar forest, in polypore fungi on *Populus* log (1, NBM); Dionne Brook P.N.A., 47.9030°N, 68.3503°W, 28.VII-9.VIII.2011, 9–23.VIII.2011, M. Roy & V. Webster, old-growth northern hardwood forest, Lindgren funnel trap (2, NBM); same locality and collectors but 47.9064°N, 68.3441°W, 27.VI-14.VII.2011, 9–23.VIII.2011, old-growth white spruce and balsam fir forest, Lindgren funnel traps (2, AFC, NBM). **Sunbury Co.**, Acadia Research Forest, 45.9866°N, 66.3841°W, 28.IV-8.V.2009, 8–13.V.2009, 13–19.V.2009, 2–9.VI.2009, 29.VII-4.VIII.2009, R. Webster & M.-A. Giguère, mature (110 year-old) red spruce forest with scattered red maple and balsam fir, Lindgren funnel traps (1 ♂, 13 sex undetermined, AFC, RWC). **York Co.**, Charters Settlement, 45.8395°N, 66.7391°W, 25–29.IV.2008, R. P. Webster, mixed forest, Lindgren funnel trap (1 ♂, RWC); 15 km W of Tracy, off Rt. 645, 45.6848°N, 66.8821°W, 25.IV-4V.2009, 4–11.V.2009, 11–19.V.2009, R. Webster & M.-A. Giguère, mature (120–180 year-old) red pine forest, Lindgren funnel traps (3 ♂, 4 sex undetermined, AFC, RWC); 14 km WSW of Tracy, S of Rt. 645, 45.6741°N, 66.8661°W, 26.IV-10.V.2010, R. Webster & C. MacKay, coll., old mixed forest with red and white spruce, red and white pine, balsam fir, eastern white cedar, red maple, and *Populus* sp., Lindgren funnel traps (2, AFC).

####### Collection and habitat data.

Little is known about the habitat associations of this species. Smetana (1973) reported a specimen from *Polyporus betulinus* (Bull.) Fr. In New Brunswick, one specimen was collected from under bark of a large, dead, standing basswood covered with bracket fungi (polypore fungi), one from under bark of a spruce log, and another in a polypore fungi on a *Populus* log. Many specimens were captured in Lindgren funnel traps deployed in a variety of forest types, including an old red pine (*Pinus resinosa* Ait.) forest, a hardwood with sugar maple (*Acer saccharum* Marsh.) and American beech (*Fagus grandifolia* Ehrh.), an old-growth northern hardwood forest, a red spruce forest, an old-growth white spruce and balsam fir forest, a mixed forest, and an old eastern white cedar forest. These traps mimic tree trunks (Lindgren 1983), and it is possible that this species lives in microhabitats associated with standing trees. Adults were collected in April, May, June, July, August, and September.

####### Distribution in Canada and Alaska.

ON, QC, NB, NS (Smetana 1971a, 1973, 1978; Bishop et al. 2009). This species was previously known in New Brunswick from one specimen collected in Dalhousie during 1925 by Johansen (Smetana 1971a).

**Map 14.**
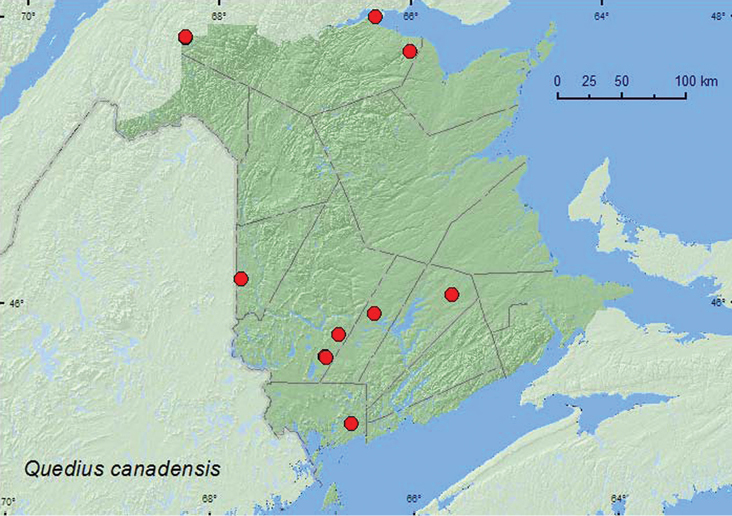
Collection localities in New Brunswick, Canada of *Quedius canadensis*.

###### Quedius (Microsaurus) criddlei

(Casey, 1915)**

http://species-id.net/wiki/Quedius_criddlei

Map 15

####### Material examined.

**New Brunswick, Queens Co.**, Cranberry Lake P.N.A., 46.1125°N, 65.6075°W, 7–13.VII.2011, 20.VII-4.VIII.2011, M. Roy & V. Webster, old red oak forest, Lindgren funnel trap (2 ♀, NBM, RWC). **Restigouche Co.**, Dionne Brook P.N.A., 47.9030°N, 68.3503°W, 27.VI-14.VII.2011, 9–23.VIII.2011, M. Roy & V. Webster, old-growth northern hardwood forest, Lindgren funnel traps (2 ♂, 2 ♀, NBM, RWC); same locality and collectors but 47.9064°N, 68.3441°W, 31.V-15.VI.2011, 15–27.VI.2011, 27.VI-14.VII.2011, 14–28.VII.2011, 9–23.VIII.2011, old-growth white spruce and balsam fir forest, Lindgren funnel traps (3 ♂, 6 ♀, AFC, NBM, RWC). **York Co.**, 15 km W of Tracy off Rt. 645, 45.6848°N, 66.8821°W, 1–8.VI.2009, R. Webster & M.-A. Giguère, old red pine forest, Lindgren funnel trap (1 ♀, RWC); same locality and habitat data, 4–16.VI.2010, R. Webster & C. MacKay, Lindgren funnel traps (1 ♂, RWC). **Quebec, Saguenay Co.** Sept-Iles, 20.V.1985, 13.IX.1985, C. Chantal (1♂, 1 ♀, CCC).

####### Collection and habitat data.

Little is known about the habitat associations of this species. Adults have been taken from leaf litter in *Larix* forests, in rotten Douglas-fir (*Pseudotsuga taxifolia* Britton) and grand fir (*Abies grandis* (Douglas ex. D. Don) Lindl.) logs, under board in a meadow, ex fungus, and in association with the ant *Formica neorufibarbis* Emery (probably accidentally) (Smetana 1971a). The New Brunswick specimens were collected in Lindgren funnel traps deployed in an old (120- to 180-year-old trees) red pine forest, an old red oak forest, an old-growth northern hardwood forest, and an old-growth white spruce and balsam fir forest. Adults were collected during May, June, July, August, and September in New Brunswick and Quebec.

####### Distribution in Canada and Alaska.

NT, YT, BC, AB, MB, ON, **QC,** **NB** (Smetana 1971a, 1990). The records from Quebec and New Brunswick represent significant range extensions east of the known distribution of this species reported by Smetana (1971a, 1990), indicating that this species is transcontinental in distribution in Canada.

**Map 16.**
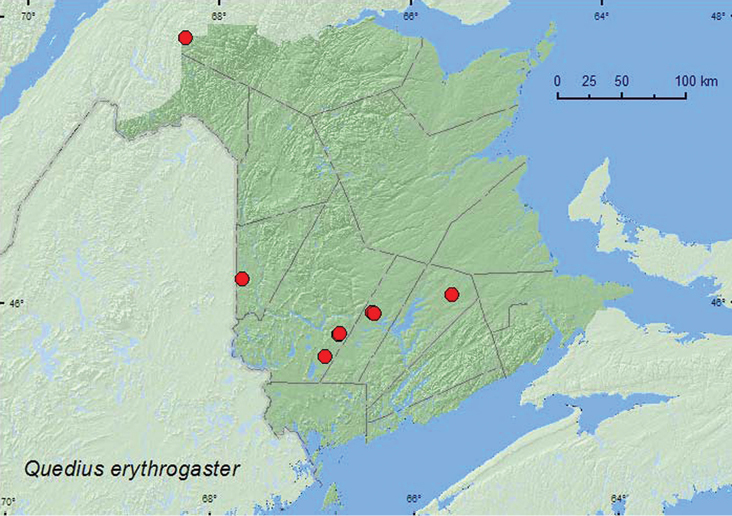
Collection localities in New Brunswick, Canada of *Quedius erythrogaster*.

###### Quedius (Microsaurus) erythrogaster

Mannerheim, 1852

http://species-id.net/wiki/Quedius_erythrogaster

Map 16

####### Material examined.

**Additional New Brunswick records, Carleton Co.**, Jackson Falls, Bell Forest, 46.2208°N, 67.7211°W, 10.IV.2005, R. P. Webster, mature hardwood forest, in leaf litter at base of tree (1 ♂, RWC). **Queens Co.**, Cranberry Lake P.N.A., 46.1125°N, 65.6075°W, 24.IV–5 V.2009, 5–12 V.2009, R. Webster & M.-A. Giguère, mature red oak forest, Lindgren funnel traps (2, AFC). **Restigouche Co.**, Dionne Brook P.N.A., 47.9030°N, 68.3503°W, 27.VI–14.VII.2011, M. Roy & V. Webster, old-growth northern hardwood forest, Lindgren funnel traps (1 ♂, 2 ♀, NBM, RWC). **Sunbury Co.**, Noonan, 45.9923°N, 66.4099°W, 22.VI.2007, S. Makepeace & R. Webster, mature mixed forest, in nest contents of barred owl, 7 m high in cavity in a red maple (1 ♂, 1 ♀, RWC); Acadia Research Forest, 45.9866°N, 66.3841°W, 22–25.IV.2009, 25.IV-4.V.2009, 4–11.V.2009, 19–25.V.2009, R. Webster & M.-A. Giguère, mature (110-year-old) red spruce forest with scattered red maple and balsam fir, Lindgren funnel traps (4, AFC). **York Co.**, Charters Settlement, 45.8395°N, 66.7391°W, 18.IV.2004, 30.IV.2004, 5.V.2006, 9.V.2006, R. P. Webster, mixed forest, in compost (decaying vegetables) (2 ♂, 2 ♀, RWC); same locality but 45.8430°N, 66.7275°W, 6.X.2005, R. P. Webster, regenerating mixed forest, baited with pile of decaying mushrooms (2 ♂, RWC); 15 km W of Tracy off Rt. 645, 45.6848°N, 66.8821°W, 22–25.IV.2009, 25.IV–4.V.2009, 4–11.V.2009, 19–25.V.2009, R. Webster & M.-A. Giguère, Lindgren funnel traps (4, AFC).

####### Collection and habitat data.

*Quedius erythrogaster*typically lives in nests and burrows of various mammals and in caves but has been found in decaying organic matter and debris (Smetana 1971a), including wet leaf litter **(**Smetana 1976). In New Brunswick, adults were found in leaf litter early in the season when snow was still present (possibly an overwinter site), compost (decaying vegetables), and decaying mushrooms. Other adults were collected from the nest contents of a barred owl in a tree hole, suggesting this species may also live in association with tree-cavity-nesting birds. Adults were also captured in Lindgren funnels traps deployed in an old red oak forest, an old red pine forest, and an old-growth northern hardwood forest. Adults were collected in April, May, June, July, and October.

####### Distribution in Canada and Alaska.

BC, AB, SK, ON, QC, NB (Smetana 1971a, 1973, 1976, 1981). *Quedius erythrogaster* was listed as occurring in New Brunswick by Majka et al. (2011) without any supporting references or data. Here, we provide the first documented records from New Brunswick.

**Map 15.**
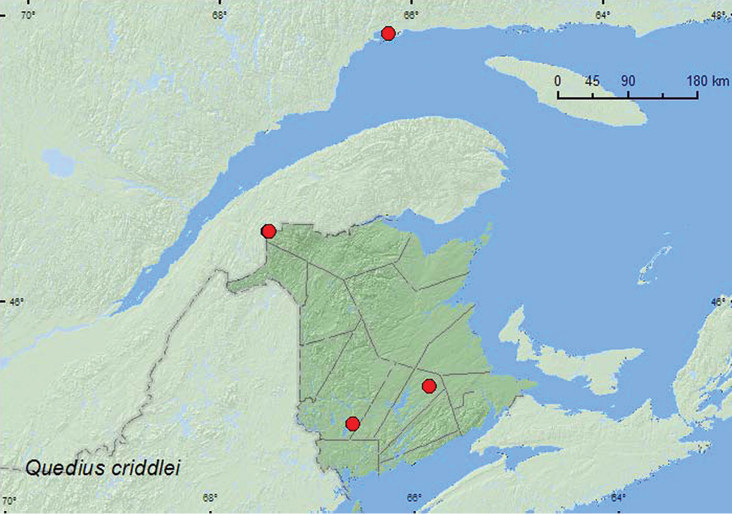
Collection localities in New Brunswick and Quebec, Canada of *Quedius criddlei*.

###### Quedius (Microsaurus) mesomelinus

(Marsham, 1802)

http://species-id.net/wiki/Quedius_mesomelinus

Map 17

####### Material examined.

**Additional New Brunswick records, Carleton Co.**, Jackson Falls, Bell Forest, 46.2200°N, 67.7231°W, 18.IX.2006, 9.X.2006, R. P. Webster, mature hardwood forest, under bark of large dead standing basswood covered with bracket fungi (1 ♂, 1 ♀, RWC); same locality, 4–12.VI.2008, 12–19.VI.2008, 19–27.VI.2008, 5–12.VII.2008, 12–19.VII.2008, 19–28.VII.2008, R. P. Webster, mature hardwood forest, Lindgren funnel traps (2 ♂, 4 ♀, 7 sex undetermined, AFC, RWC); same locality, 14–20.V.2009, 16–21.VI.2009 , R. Webster & M.-A. Giguère, mature hardwood forest, Lindgren funnel traps (2, AFC). **Restigouche, Co.**, Dionne Brook P.N.A., 47.9064°N, 68.3441°W, 27.VI-14.VII.2011, M. Roy & V. Webster, old-growth white spruce and balsam fir forest, Lindgren funnel trap (1, NBM). **York Co.**, Odell Park, 45.9570°N, 66.6695°W, 19.VI.2005, R. P. Webster, moist wood chips and decaying plant material (1 ♀, RWC).

####### Collection and habitat data.

In Europe, this species is typically found in synanthropic situations in decaying organic materials such as compost (Smetana 1971a). It has also been reported from mammal burrows, tree holes, and caves in natural settings. In New Brunswick, adults were collected from under bark of a fungus-covered, dead, standing basswood, among moist wood chips and decaying plant material, and from Lindgren funnel traps deployed in a hardwood forest and an old-growth white spruce and balsam fir forest. Adults were captured in June, July, September, and October.

####### Distribution in Canada and Alaska.

AK,BC, AB, MB, ON, QC, NB, NS, NF (Smetana 1971a; Majka and Smetana 2007). This adventive species was first reported from New Brunswick by Majka and Smetana (2007) from specimens collected in Saint John in 1907 by G. Morrisey.

**Map 17.**
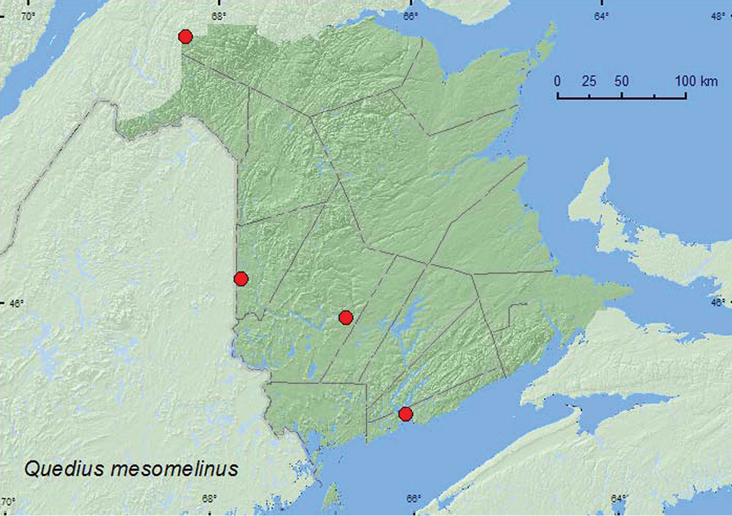
Collection localities in New Brunswick, Canada of *Quedius mesomelinus*.

###### Quedius (Microsaurus) peregrinus

Gravenhorst, 1806

http://species-id.net/wiki/Quedius_peregrinus

Map 18

####### Material examined.

**Additional New Brunswick records. Albert Co.**, Caledonia Gorge P.N.A., 45.8257°N, 64.7791°W, 6.VII.2011, R. P. Webster, old hardwood forest (sugar maple and beech), in decayed fleshy polypore in log (1 ♂, NBM). **Sunbury Co.**, Maugerville, Portobello Creek N.W.A., 45.9031°N, 66.4268°W, 11.IX.2006, R. P. Webster, red oak and red maple forest, on gilled mushroom (1 ♀, RWC); Acadia Research Forest, pitfall trap (collection) dates, 17.VIII.1999, 24.VIII.1999, 25.VIII.1999, 8.IX.1999, 13.IX.1999, 15.IX.1999, G. Gesner, strip, select., and control plots (15, AFC); same locality but 46.0188°N, 66.3765°W, 17.VIII.2007, R. P. Webster, mature red spruce and red maple forest, sifting moss (1, AFC); same locality and collector but 46.0173°N, 66.3741°W, 18.IX.2007, 8.5 year-old regenerating mixed forest, in sphagnum and leaf litter at bottom of old tire depression (1, AFC). **York Co.**, Charters Settlement, 45.8395°N, 66.7391°W, 18.VII.2006, R. P. Webster, mixed forest, on rotting fungus covered log (1 ♀, RWC); same locality data, collector, and (adjacent) forest type, 4.X.2005, residential lawn, on soil at base of grasses (1 ♂, RWC); same locality, collector, and adjacent forest type but 45.8348°N, 66.7335°W, 4.VIII.2004, in fleshy fungi (1, NBM); same locality and collector but 45.8430°N, 66.7275°W, 12.VII.2005, regenerating mixed forest, beating foliage (2 ♂, 3 ♀, RWC); same locality data, collector, and forest type, 25.IX.2005, baited with pile of decaying mushrooms (1 ♂, RWC).

####### Collection and habitat data.

One of the Nova Scotia specimens was collected by treading wet *Sphagnum* (Smetana 1973), otherwise little is known about the habitat associations of this species (Smetana 1971a). Specimens from New Brunswick were collected in red oak and red maple forests, a red spruce and red maple forest, an old sugar maple and American beech forest, and regenerating mixed forests. Adults were collected from gilled mushrooms, a decayed fleshy polypore fungus on a log, from a rotten fungus-covered log, baited with decaying mushrooms, sifted from sphagnum and leaf litter at bottom of old tire depression, and swept from foliage in a regenerating forest. Adults were also captured in pitfall traps in large numbers (Klimaszewski et al. 2005). This species is probably associated with decaying organic matter, such as decaying mushrooms. Adults were captured during July, August, September, and October.

####### Distribution in Canada and Alaska.

ON, QC, NB, NS (Smetana 1971a, 1973; Klimaszewski et al. 2005). This species was first reported from New Brunswick by Klimaszewski et al. (2005) from specimens collected in pitfall traps at the Acadia Research Forest (Sunbury Co.)

**Map 18.**
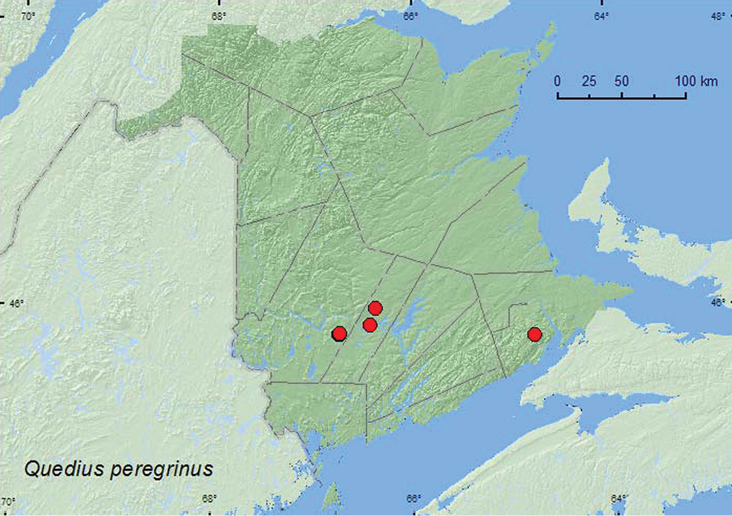
Collection localities in New Brunswick, Canada of *Quedius peregrinus*.

###### Quedius (Quedius) curtipennis

Bernhauer, 1908

http://species-id.net/wiki/Quedius_curtipennis

Map 19

####### Material examined.

**Additional New Brunswick records. Carleton Co.**, Jackson Falls, Bell Forest, 46.2152°N, 67.7190°W, 21.VIII.2004, 15.IX.2004, R. P. Webster, upper river margin under litter on clay soil (2 ♀, RWC); Hovey Hill P.N.A., 46.1115°N, 67.7770°W, 10.V.2005, R. P. Webster, mature hardwood forest in moist leaf litter and moss on margin of vernal pond (2 ♂, 1♀, RWC). **York Co.**, Pokiok, 2.VI.1995, (G. Gesner) pitfall trap (1♂, AFC); Charters Settlement, 45.8395°N, 66.7391°W, 30.IV.2005, R. P. Webster, mixed forest, in compost (decaying vegetables) (1 ♂, RWC); same locality data, collector, and forest type, 5.IX.2006, in pile of decaying corncobs and cornhusks (1♀, RWC).

####### Collection and habitat data.

This species has been reported mostly from around human settlements in various kinds of debris and under stones (Smetana (1971a). Adults have also been found in natural habitats in moss and leaf litter (Smetana 1971a, 1978). In New Brunswick, adults were collected in natural habitats (river margin in litter, in moist leaf litter and moss on vernal pond margin in a hardwood forest) and in synanthropic situations (in compost and pile of decaying corncobs and cornhusks near a home). Adults were collected in April, May, June, August, and September.

####### Distribution in Canada and Alaska.

BC, ON, NB, NS (Smetana 1971a; Majka and Smetana 2007; Brunke and Marshall 2011). This adventive species was first reported from New Brunswick and Nova Scotia (and eastern North America) by Majka and Smetana (2007). An earlier record (Truro, 1984) from Nova Scotia was later reported by Majka and Klimaszewski (2008a). The record from New Brunswick was based on a specimen collected by CG Majka at Mary’s Point in 2002.

**Map 19.**
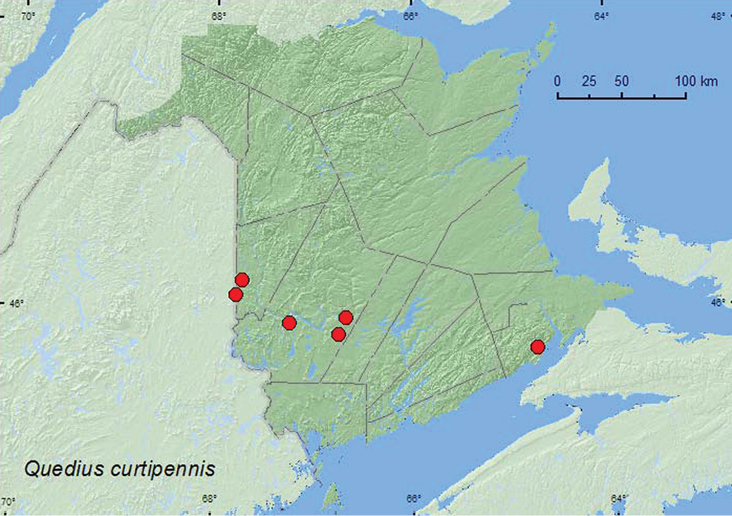
Collection localities in New Brunswick, Canada of *Quedius curtipennis*.

###### Quedius (Quedius) labradorensis labradorensis

Smetana, 1965

http://species-id.net/wiki/Quedius_labradorensis_labradorensis

Map 20

####### Material examined.

**Additional New Brunswick records, Restigouche Co.**, Little Tobique River near Red Brook, 47.4462°N, 67.0689°W, 24.V.2007, R. P. Webster, coll., old growth eastern white cedar forest, in moss and leaf litter near brook (1 ♂, NBM); MacFarlane Brook P.N.A., 47.6018°N, 67.6263°W, 25.V.2007, R. P. Webster, old growth eastern white cedar swamp, in moss near brook (1 ♂, RWC); Berry Brook P.N.A., 47.8140°N, 66.7578°W, 26.V.2007, R. P. Webster, old growth eastern white cedar swamp, in moss on hummock at base of eastern white cedar (2 ♀, RWC). **Saint John Co.**, Musquash, 45.1696°N, 66.3140°W, 7.V.2006, R. P. Webster, spruce forest, in sphagnum and litter on margin of brook (1 ♂, RWC); ca. 2.0 km NE of Maces Bay, 45.1168°N, 66.4552°W, 8.V.2006, R. P. Webster, eastern white cedar swamp, under deer dung (1 ♂, RWC). **York Co.,** Charters Settlement, 45.8395°N, 66.7391°W, 30.IV.2004, R. P. Webster, mixed forest, m.v. light (1 ♀, RWC).

####### Collection and habitat data.

Smetana (1976) reported this species from moss, mushrooms, and deciduous leaf litter (birch and poplar stands) near streams. In the Alberta foothills forests, *Quedius labradorensis* was considered to be an open-ground specialist after forest harvesting and only extended a short distance into adjacent uncut forests (Pohl et al. (2007). Numbers of this species also increased after harvesting. In New Brunswick, most adults were found in old-growth eastern white cedar swamps in moss usually near small streams. One adult was found under white-tailed deer (*Odocoileus virginianus* (Zimmerman)) dung, and another was collected at a mercury-vapor light. Adults were collected during April and May.

####### Distribution in Canada and Alaska.

AK, BC, NT, AB, MB, ON**,** QC, NB, NF (Smetana 1971a, 1973, 1976, 1978). Majka et al. (2011) listed this species as occurring in New Brunswick without any supporting references or data. Here, we provide the first documented records from New Brunswick.

**Map 20.**
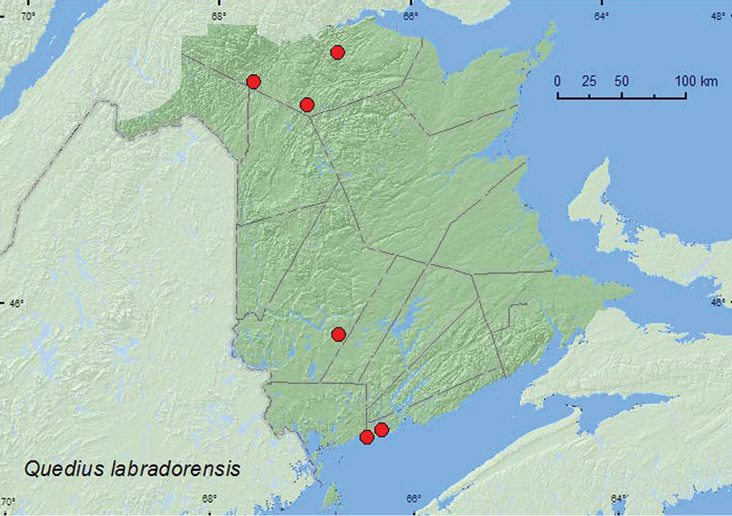
Collection localities in New Brunswick, Canada of *Quedius l. labradorensis*.

###### Quedius (Quedionuchus) plagiatus

Mannerheim, 1843

http://species-id.net/wiki/Quedius_plagiatus

Map 21

####### Material examined.

**Additional New Brunswick records, Albert Co.**, Caledonia Gorge P.N.A., 45.8257°N, 64.7791°W, 6.VII.2011, R. P. Webster, old hardwood forest (sugar maple and beech), under bark of sugar maple log (1, NBM). **Carleton Co.**, Hovey Hill P.N.A., 46.1115°N, 67.7770°W, 19.VIII.2004, R. P. Webster, hardwood forest, under bark of log (1 ♀, RWC); Jackson Falls, Bell Forest, 46.2200°N, 67.7231°W, 4–12.VI.2008, R. P. Webster, mature hardwood forest, Lindgren funnel trap (1, AFC); same locality, 14–20.V.2009, 16–21.VI.2009 , R. P. Webster & M.-A. Giguère, mature hardwood forest, Lindgren funnel traps (2, AFC). **Charlotte Co.**, 5.1 km NW of Pomeroy Ridge, 45.3055°N, 67.4340°W, 5.VI.2008, R. P. Webster, mixed forest, under bark of spruce log (1, NBM); 10 km NW of New River Beach, 45.2110°N, 66.6170°W, 30.IV-17.V.2010, R. Webster & V. Webster, coll., old growth eastern white cedar forest, Lindgren funnel trap (1, AFC). **Queens Co.**, Cranberry Lake P.N.A., 46.1125°N, 65.6075°W, 24.IV-5 V.2009, 21–27.V.2009, 5–11.VI.2009, 18–25.VI.2009, 25.VI-1 VII.2009, 21–28.VII.2009, 6–14.VIII.2009, R. Webster & M.-A. Giguère, mature red oak forest, Lindgren funnel traps (2 ♀, 13 sex undetermined, AFC, NBM, RWC). **Restigouche Co.**, Little Tobique River near Red Brook, 47.4462°N, 67.0689°W, 24.V.2007, R. P. Webster, old-growth eastern white cedar forest, under bark of large fallen spruce (1 ♀, NBM); Jacquet River Gorge P.N.A., 47.8200°N, 66.0015°W, 13.V.2010, R. P. Webster (1, NBM); same locality but 47.8257°N, 66.0779°W, 14.V.2010, P. Giasson, old mixed forest, under bark of *Populus* sp. log (1, NBM); South Branch Rd., 494 m elev., 47.8767°N, 68.2657°W, 22.VI.2010, R. P. Webster, Spruce and balsam fir forest, under bark of spruce (1, NBM); Dionne Brook P.N.A., 47.9030°N, 68.3503°W, 28.VII-9.VIII.2011, M. Roy & V. Webster, old-growth northern hardwood forest, Lindgren funnel trap (2, AFC, NBM); same locality and collectors but 47.9064°N, 68.3441°W, 15–27.VI.2011, old-growth white spruce and balsam fir forest, Lindgren funnel traps (2, AFC, NBM). **Sunbury Co.**, Acadia Research Forest, 45.9866°N, 66.3841°W, 6–24.VI.2009, 13–21.VII.2009, 21–29.VII.2009, 29.VII-4.VIII.2009, R. Webster & M.-A. Giguère, mature (100 year-old) red spruce forest with scattered red maple and balsam fir, Lindgren funnel trap (4, AFC). **York Co.**, Charters Settlement, 45.8188°N, 66.7460°W, 25.VIII.2004, R. P. Webster, clear-cut, under bark of conifer stump (3 ♂, 3 ♀, RWC); same locality but, 45.8380°N, 66.7310°W, 14.V.2004, R. P. Webster, mixed forest, under bark of conifer (1 ♀, RWC); 15 km W of Tracy, off Rt. 645, 45.6848°N, 66.8821°W, 8–15.VI.2009, 21–28.VI.2009, R. Webster & M.-A. Giguère, mature (120–180 year-old) red pine forest, Lindgren funnel traps (4, AFC); 14 km WSW of Tracy, S of Rt. 645, 45.6741°N, 66.8661°W, 26.IV-10.V.2010, R. Webster & C. MacKay, old mixed forest with red and white spruce, red and white pine, balsam fir, eastern white cedar, red maple, and *Populus* sp., Lindgren funnel trap (1, AFC).

####### Collection and habitat data.

This common Holarctic species is found under bark of dead trees, usually coniferous species (Smetana 1971a). In New Brunswick, most specimens were found under bark of conifer logs or stumps and some under bark of hardwoods. Adults were also commonly captured in Lindgren funnel traps. Adults were collected in April, May, June, July, and August.

####### Distribution in Canada and Alaska.

AK, YT, NT, BC, AB, SK, MB, ON, QC, NB, NS (Smetana 1971a; Bishop et al. 2009). *Quedius plagiatus* was listed as occurring in New Brunswick by Majka et al. (2011) without any supporting references or data. Here, we provide the first documented records from New Brunswick.

**Map 21.**
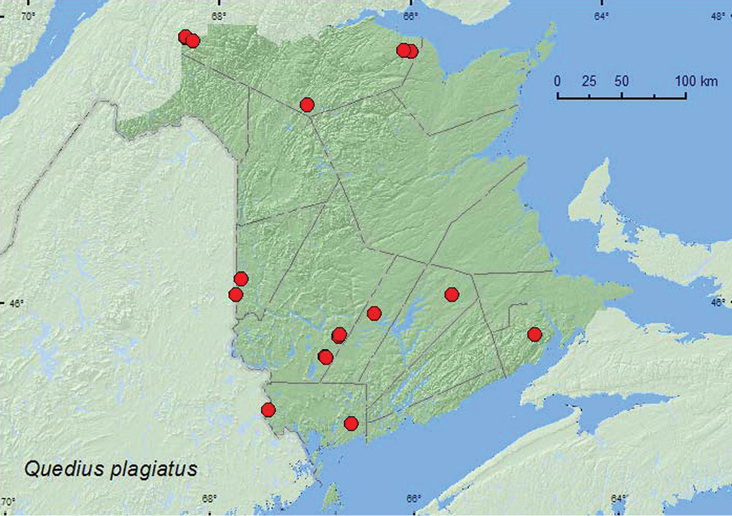
Collection localities in New Brunswick, Canada of *Quedius plagiatus*.

###### Quedius (Distichalius) capucinus

(Gravenhorst, 1806)

http://species-id.net/wiki/Quedius_capucinus

Map 22

####### Material examined.

**New Brunswick,** **Queens Co.**, Cranberry Lake P.N.A., 46.1125°N, 65.6075°W, 10–11.VII.2009, R. Webster & M.-A. Giguère, mature red oak forest, u.v. light (2 ♂, 1 ♀, AFC). **Restigouche Co.**, Dionne Brook P.N.A., 47.9030°N, 68.3503°W, 14–28.VII.2011, 28.VII-9.VIII.2011, M. Roy & V. Webster, old-growth northern hardwood forest, Lindgren funnel traps (2, AFC, NBM). **York Co.**, Charters Settlement, 45.8430°N, 66.7275°W, 14.IX.2004, 17.IX.2004, 25.IX.2004, 6.X.2005, R. P. Webster, regenerating mixed forest, baited with pile of decaying mushrooms (1 ♂, 5 ♀, NBM, RWC); same locality but 45.8286°N, 66.7365°W, 24.VI.2006, R. P. Webster, mature mixed forest, in gilled mushroom (1 ♂, RWC); same locality but 45.8395°N, 66.7391°W, 28.IX.2006, 29.VIII.2007, R. P. Webster, mixed forest, in pile of corncobs and cornhusks (1 ♂, 1 ♀, RWC).

####### Collection and habitat data.

This species appears to prefer decaying organic substances and has been found on carrion, under human feces (Smetana 1971a), and in fleshy fungi (Blatchley 1910). In New Brunswick, most specimens were found in decaying mushrooms and compost (pile of corncobs and cornhusks). Two individuals were captured in Lindgren funnel traps deployed in an old-growth northern hardwood forest. Adults were found in April, June, July, August, September, and October.

####### Distribution in Canada and Alaska.

ON, QC, **NB**, NS (Smetana 1971a; Bishop et al. 2009).

**Map 22.**
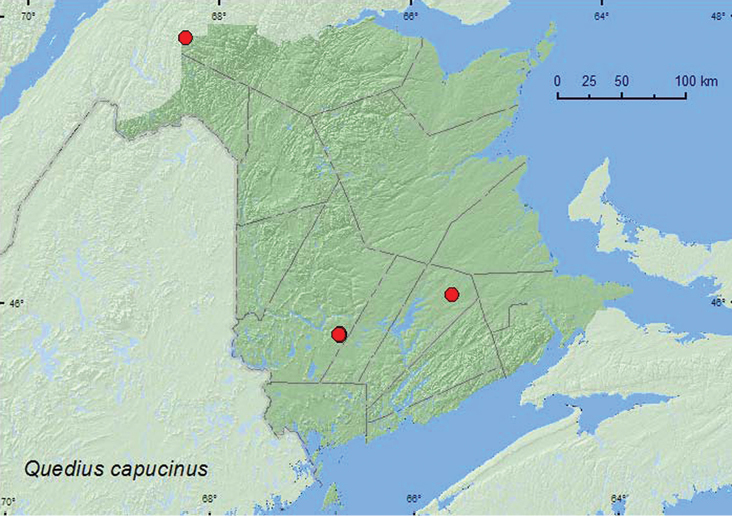
Collection localities in New Brunswick, Canada of *Quedius capucinus*.

###### Quedius (Distichalius) cinctus

(Paykull, 1790)

http://species-id.net/wiki/Quedius_cinctus

Map 23

####### Material examined.

**Additional New Brunswick records. Restigouche Co.**, Mount Atkinson, 447 m elev., 47.8192°N, 68.2618°W, 21.VII.2010, R. P. Webster, spruce and balsam fir forest (boreal forest), small shaded spring-fed brook with mossy margin, in wet moss (1 ♂, RWC). **York Co.**, Charters Settlement, 45.8430°N, 66.7275°W, 8.X.2004, 6.X.2005, R. P. Webster, regenerating mixed forest, baited with pile of decaying mushrooms (1 ♂, 3 ♀, NBM, RWC); Charters Settlement, 45.8395°N, 66.7391°W, 18.X.2004, 6.IX.2005, 5.VIII.2005, 29.III.2006, 17.IX.2006, 26.IX.2007, R. P. Webster, mixed forest, in pile of decaying (moldy) corncobs and cornhusks (4 ♂, 3 ♀, NBM, RWC); same locality data and collector but 17.IX.2006, mixed forest, in pile of decaying leaves (1 ♂, RWC).

####### Collection and habitat data.

In Europe, *Quedius cinctus*usually occurs in decaying organic material, usually near human settlements (Smetana 1971a). In New Brunswick, specimens were similarly found in decaying organic material (decaying mushrooms, decaying corncobs and cornhusks, decaying leaves). One individual was found in wet moss along a cold, shaded brook. Adults were collected in April, July, September, and October.

####### Distribution in Canada and Alaska.

ON, NB (Majka et al. 2009; Brunke and Marshall 2011). This adventive species was first reported from New Brunswick and Canada by Majka et al. (2009) from three specimens collected from a domestic pig carcass in Bouctouche (Kent Co.) in 2007. This species is probably well established in New Brunswick and likely occurs in the intervening areas between this province and Massachusetts (Framingham and Fall River, USA), where the species was first reported by Smetana (1971a) from North America.

**Map 23.**
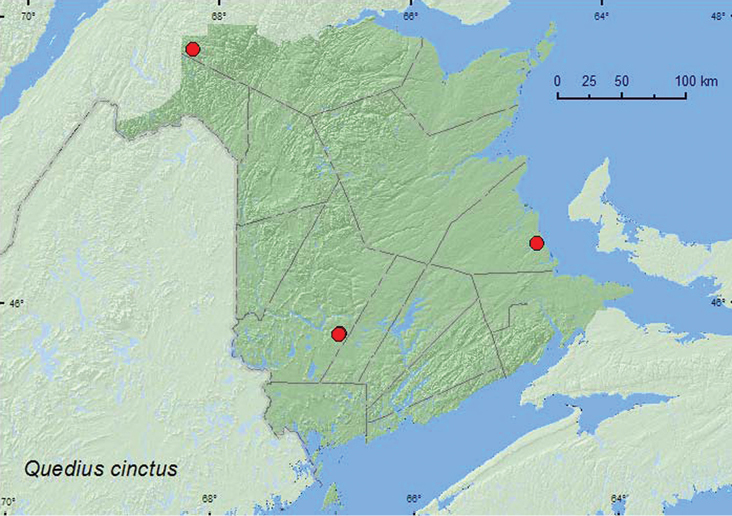
Collection localities in New Brunswick, Canada of *Quedius cinctus*.

###### Quedius (Raphirus) frigidus

Smetana, 1971**

http://species-id.net/wiki/Quedius_frigidus

Map 24

####### Material examined.

**New Brunswick, Restigouche Co.**, Berry Brook P.N.A., 47.8140°N, 66.7578°W, 26.V.2007, R. P. Webster, old-growth eastern white cedar forest in moss and leaf litter near brook (4 ♂, 4 ♀, NBM, RWC); MacFarlane Brook P.N.A., 47.6018°N, 67.6263°W, 25.V.2007, R. P. Webster, old growth eastern white cedar forest in moss near brook (4 ♂, NBM, RWC); Mount Atkinson, 447 m elev., 47.8192°N, 68.2618°W, 23.VI.2010, R. P. Webster, spruce and balsam fir forest (boreal forest), small, shaded, spring-fed brook with mossy margin, in wet moss (1 ♂, RWC).

####### Collection and habitat data.

Relatively little was previously known about the habitat requirements of this species, although it appears that this species may be associated with moss and leaf litter near small streams and other wetlands. Smetana (1973, 1976) reported the species from leaf litter in a sphagnum bog, wet sphagnum (treading) along the margin of a large spring, and in leaf litter (sifting) along a small stream. In the Alberta Foothills forests, *Quedius frigidus* was considered to be a mature forest specialist (Pohl et al. 2007). Adults at the three New Brunswick localities were found in moss and leaf litter near brooks in old-growth eastern white cedar forests and a mature spruce and balsam fir forest on the north-facing slope of a hill. Adults were collected during May and June.

####### Distribution in Canada and Alaska.

AK, NT, ON, **NB**, NF (Smetana 1973, 1976, 1978, 1981). Smetana (1971a, 1973) suggested that *Quedius frigidus* was a northern transcontinental species with glacial relic populations in southern areas at higher mountain elevations. The New Brunswick records indicate a more southerly distribution at low elevations in eastern Canada.

**Map 24.**
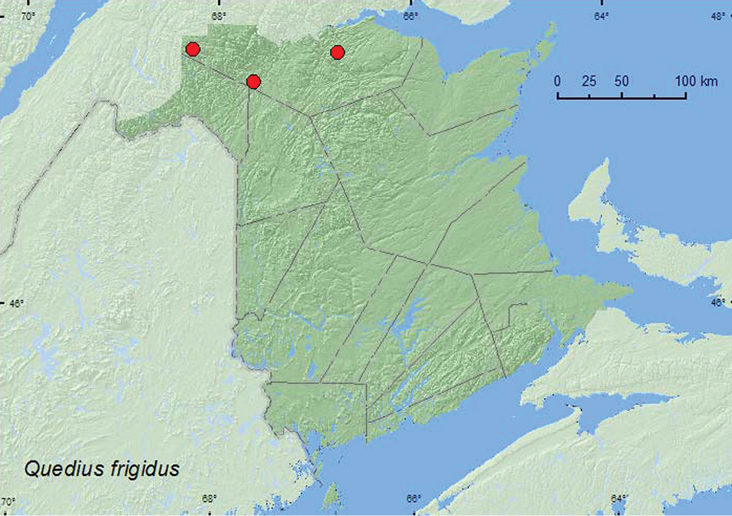
Collection localities in New Brunswick, Canada of *Quedius frigidus*.

###### Quedius (Raphirus) fulvicollis

(Stephens, 1833)**

http://species-id.net/wiki/Quedius_fulvicollis

Map 25

####### Material examined.

**New Brunswick, Albert Co.**, Caledonia Gorge P.N.A., 45.8176°N, 64.7800°W, 6.VII.2011, R. P. Webster, old hardwood forest (sugar maple and beech), in mossy seepage area with *Carex*, sifting moss and grass litter (2 ♂, 1♀, NBM, RWC). **Restigouche Co.**, 7.5 km S of Saint Arthur, 47.8283°N, 66.7654°W, 14.VI.2006, R. P. Webster, old-growth eastern white cedar forest, in moist leaves at base of large white birch (1 ♂, RWC). **York Co.**, Charters Settlement, 45.8395°N, 66.7391°W, 19.V.2005, R. P. Webster, alder swamp, in moist leaf litter near small (slow flowing) brook (1 ♂, RWC); Mazerolle Settlement, 45.8717°N, 66.8273°W, 28.IV.2006, R. P. Webster, eastern white cedar swamp, in moss and leaf litter near brook (1 ♀, RWC); 8 km NW of Magundy, 45.8712°N, 67.2221°W, 8.VII.2006, R. P. Webster, mature hardwood forest, margin of small cold (spring-fed) brook among sedges (1 ♂, 2 ♀, RWC).

####### Collection and habitat data.

Adults of this species occur in moist moss, sphagnum, deciduous leaf litter, and mixed forest litter in various wet biotypes such as small stream margins, lake margins, and wet areas in forests (Smetana 1971a, 1973, 1976). In New Brunswick, most specimens were found in moist leaf litter, moss, or among sedges near small brooks in alder swamps, eastern white cedar forests, and hardwood forests. Adults were collected during April, May, June, and July.

####### Distribution in Canada and Alaska.

AK, YT, BC, AB, MB, ON, QC, **NB**, NF (Smetana 1971a, 1973, 1976).

**Map 26.**
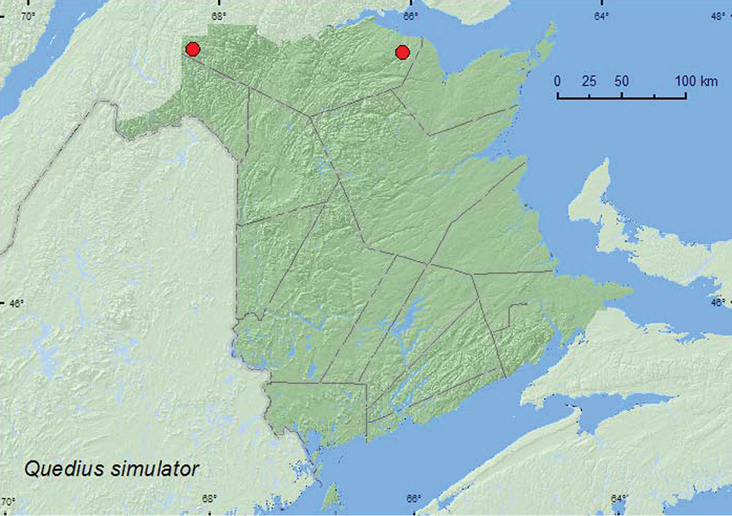
Collection localities in New Brunswick, Canada of *Quedius simulator*.

###### Quedius (Raphirus) simulator

Smetana, 1971**

http://species-id.net/wiki/Quedius_simulator

Map 26

####### Material examined.

**New Brunswick, Restigouche Co.**, Mount Atkinson, 447 m elev., 47.8192°N, 68.2618°W, 21.VII.2010, R. P. Webster, spruce and balsam fir forest (boreal forest), small, shaded, spring-fed brook with mossy margin, in wet moss (1 ♂, RWC); Jacquet River Gorge P.N.A., 47.8109°N, 66.0905°W, 13.VIII.2010, R. P. Webster, old mixed forest, small shaded spring-fed brook with mossy margin, in wet moss (1 ♂, 2 ♀, RWC).

####### Collection and habitat data.

This species was reported from very wet debris and moss in small gullies and depressions on the forest floor and edges of oligotrophic ponds in mixed forests, and from *Carex* hummocks and wet debris from various wet habitats such as lake margins, swamps, and marshes (Smetana 1971, 1973). Specimens from Moosonee, Ontario were collected by sifting leaf litter under willow (*Salix* sp.) and alder bushes near the Moose River (Smetana 1976). In New Brunswick, adults were found in wet moss along shaded, cold, spring-fed brooks. Adults were collected during July and August.

####### Distribution in Canada and Alaska.

AK, NT, BC, AB, SK, MB, ON, QC, **NB,** LB (Smetana 1971a, 1973, 1976, 1981, 1990). This is a northern transcontinental species, with most records from the boreal forest of northern Canada (Smetana 1971a, 1973, 1976, 1981).

**Map 25.**
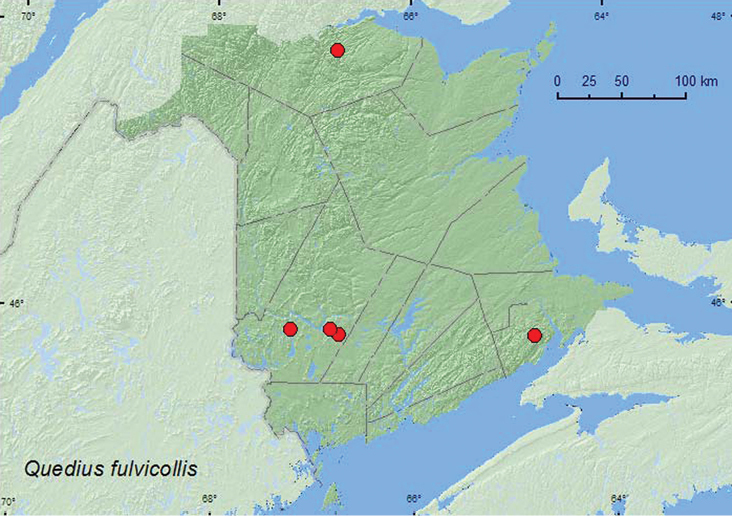
Collection localities in New Brunswick, Canada of *Quedius fulvicollis*.

##### Subtribe Staphylinina Latreille, 1802

###### Staphylinus ornaticauda

LeConte, 1863

http://species-id.net/wiki/Staphylinus_ornaticauda

Map 27

####### Material examined.

**New Brunswick, Charlotte Co.**, 3 km SW of King Brook Lake, 45.3194°N, 67.4414°W, 27.V.2007, R. P. Webster, eastern white cedar, red maple, and black ash swamp, in moist litter and moss near small pools with *Carex* (2 ♂, 1 ♀, RWC); 3.5 km NW of Pomeroy Ridge, 45.3087°N, 67.4362°W, 5.VI.2008, 16.VI.2008, R. P. Webster, red maple swamp, in leaves and moss near small vernal pool with *Carex* (1 ♂, 2 ♀, NBM). **Northumberland Co.**, Goodfellow Brook P.N.A., 46.8943°N, 65.3796°W, 23.V.2007, R. P. Webster, old-growth eastern white cedar swamp, in moss and litter on hummock (2 ♂, RWC). **York Co.**, Canterbury, “Browns Mountain Fen”, 45.8967°N, 67.6343°W, 2.V.2005, 29.IV.2006, M.-A. Giguère & R. P. Webster, eastern white cedar swamp, in litter at base of cedar (2 ♂, RWC); same locality but 45.8957°N, 67.6462°W, 29.IV.2006, R. P. Webster, eastern white cedar swamp in sphagnum and litter near vernal pool with *Carex* (1 ♂, 1 ♀, RWC).

####### Collection and habitat data.

Brunke et al. (2011) reported that *Staphylinus ornaticauda* was restricted to wetlands, such as bogs and fens, with an abundance of sphagnum moss. In New Brunswick, this species appears to be associated with eastern white cedar swamps and fens and red maple swamps on calcareous soils. Adults were collected in eastern white cedar swamps, an eastern white cedar, red maple, and black ash (*Fraxinus nigra* Marsh.) swamp, and in a red maple swamp near an eastern white cedar swamp. Adults occurred in litter at the base of cedars, and in moist litter and moss near small vernal pools with *Carex*. Adults were collected by sifting litter. This species was collected in April, May, and June. This species is flightless (Brunke et al. 2011). Due to the limited dispersal capabilities, restricted habitat requirements, and apparent rarity, Brunke et al. (2011) suggested that this species should be studied as a potential species at risk.

####### Distribution in Canada and Alaska.

MB, ON, PQ, **NB**, NS (Campbell and Davies 1991, as *Staphylinus erythropterus* Linnaeus).

**Map 27.**
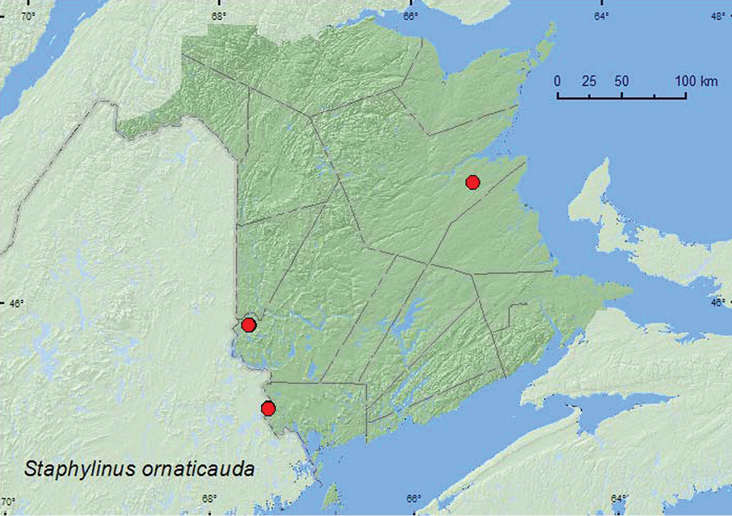
Collection localities in New Brunswick, Canada of *Staphylinus ornaticuada*.

##### Subtribe Philonthina Kirby, 1837

###### Bisnius cephalicus

Casey, 1915**

http://species-id.net/wiki/Bisnius_cephalicus

Map 28

####### Material examined.

**New Brunswick, Restigouche Co.**, 7.5 km S of Saint Arthur, 47.8283°N, 66.7654°W, 14.VI.2006, R. P. Webster, old-growth eastern white cedar forest in moist leaves at base of large white birch (1 ♂, 1 ♀, NBM, RWC); Berry Brook P.N.A., 47.8140°N, 66.7578°W, 26.V.2007, R. P. Webster, old-growth eastern white cedar swamp, in moss and leaf litter under alders (1 ♂, 1 ♀, RWC); MacFarlane Brook P.N.A., 47.6018°N, 67.6263°W, 25.V.2007, R. P. Webster, old-growth eastern white cedar swamp, in moss and leaves under alders near brook (1 ♀, RWC); Jacquet River Gorge P.N.A., 47.8204°N, 66.0833°W, 14.VI.2008, R. P. Webster, river margin in drift material (1 ♂, RWC).

####### Collection and habitat data.

Little was previously known about the habitat requirements of this species. In New Brunswick, this species was found in leaf litter and moss near brooks in old-growth eastern white cedar forests and in drift material along a river margin (1). Adults were collected in May and June.

####### Distribution in Canada and Alaska.

AB, MB, ON, **NB** (Smetana 1995). This species was previously known from only three specimens from two localities, a single female from the type locality at “Aweme”, Manitoba and two specimens from George Lake, Alberta (Smetana 1995) Recently, Brunke and Marshall (2011) reported another specimen of this species from N. Moosonee, Ontario.

**Map 28.**
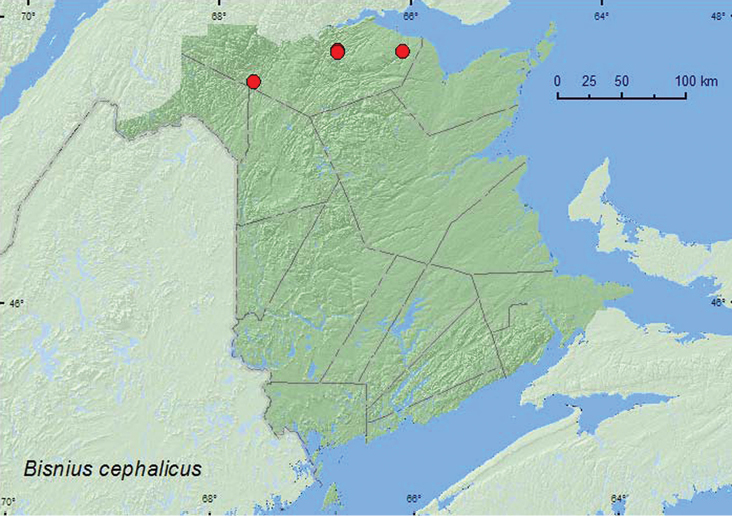
Collection localities in New Brunswick, Canada of *Bisnius cephalicus*.

###### Bisnius cephalotes

(Gravenhorst, 1802)

http://species-id.net/wiki/Bisnius_cephalotes

####### Remarks. 

*Bisnius cephalotes*was reported by Majka and Klimaszewski (2008a) from New Brunswick based on three specimens collected by R. P. Webster from New Maryland. There are no specimens of this species in the collections of R. P. Webster or C. Majka from New Brunswick. This species is, therefore, removed from the faunal list of New Brunswick.

###### Bisnius palmi

Smetana, 1955

http://species-id.net/wiki/Bisnius_palmi

Map 29

####### Material examined.

**New Brunswick, Queens Co.**, near Quarries, 45.6005°N, 66.0500°W, 25.IX.2006, S. Makepeace, contents from barred owl nest box, 8 m high on red maple (1, ♀, RWC); 4 km W of Lower Gagetown, 45.7466°N, 66.1862°W, 30.VII.2006, S. Makepeace, mixed red oak and pine forest, contents of red shouldered hawk nest 12 m high in red oak (3 ♂, 2 ♀, RWC); Pleasant Villa, 45.7023°N, 66.1732°W, 15.VI.2007, S. Makepeace & R. Webster (1, ♀, NBM); Rees, near Grand Lake, 46.0016°N, 65.9466°W, 29.V.2007, S. Makepeace & R. Webster, nest box contents of barred owl (4 ♂, 6 ♀ NBM, RWC); McAlpines near Upper Hampstead Rd., 45.7250°N, 66.1200°W, 3.VI.2007, S. Makepeace & R. Webster, nest contents of barred owl (1 ♂, 1 ♀, NBM); Cranberry Lake P.N.A., 46.1125°N, 65.6075°W, 7–22.VI.2011, 13–20.VII.2011, M. Roy & V. Webster, old red oak forest, Lindgren funnel traps in forest canopy (2 ♂, NBM). **Sunbury Co.**, Noonan, 45.9923°N, 66.4099°W, 2.VI.2007, S. Makepeace & R. P. Webster, nest contents of barred owl from tree hole 7 m high in red maple (1 ♀, NBM). **Westmorland Co.**, Sackville, near Ogden Mill, 45.9216°N, 64.3893°W, 12.V.2006, S. Makepeace, black spruce forest, in nest contents of great horned owl, *Bubo virginianus* (2 ♀, NBM, RWC). **York Co**., Graham Corner, 45.8565°N, 67.7083°W, 26.VI.2007, S. Makepeace & R. Webster, nest contents of barred owl from tree hole in sugar maple (1 ♂, 1 ♀, NBM); Marysville, 45.9750°N, 66.5700°W, 22.VI.2007, S. Makepeace & R. Webster, nest box contents of barred owl (1 ♀, NBM).

####### Collection and habitat data.

This species was reported by Smetana (1995) from various habitats associated with trees, such as swallow nests, old squirrel nests, and a red oak tree fork hole. In New Brunswick, all but one of the *Bisnius palmi* specimens were found in the nest contents of a great horned owl (*Bubo virginianus* Gmelin) and barred owls, which normally nest in tree holes or in artificial nest boxes on trees. This species was also found in the nest contents of a red shouldered hawk (*Buteo lineatus* Gmelin), which makes large nests within forks of large trees. One adult was captured in a Lindgren funnel trap deployed in the canopy of a red oak in an old red oak forest. Adults were collected in May, June, July, and September.

####### Distribution in Canada and Alaska.

ON, **NB**, NS (Smetana 1995). *Bisnius palmi* is transcontinental in North America, with most records from eastern North America. There is one record from Nova Scotia (Caribou Island). *Bisnius palmi* was originally described from Sicily, Italy, but was apparently an accidental, introduced specimen (Smetana 1995).

**Map 29.**
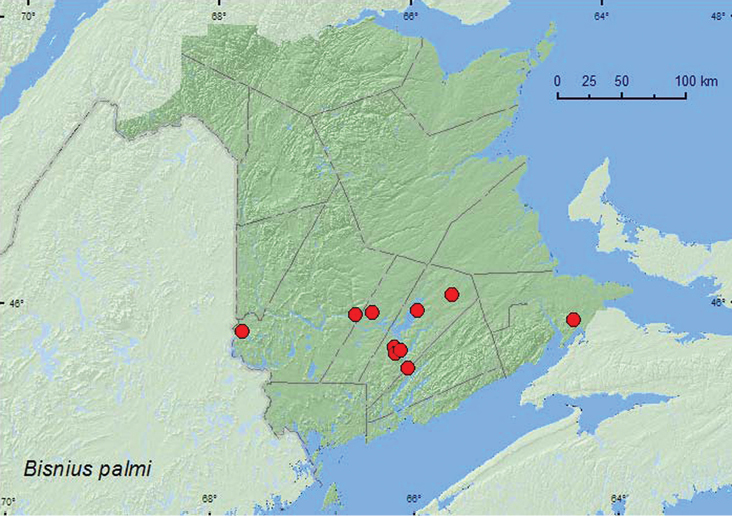
Collection localities in New Brunswick, Canada of *Bisnius palmi*.

###### Bisnius quediinus

Horn, 1884**

http://species-id.net/wiki/Bisnius_quediinus

Map 30

####### Material examined.

**New Brunswick, Queens Co.**, Cranberry Lake P.N.A., 46.1125°N, 65.6075°W, 21–27.V.2009, R. Webster & M.-A. Giguère, mature red oak forest, Lindgren funnel traps (2 ♀, 1 sex undetermined, AFC, RWC); Grand Lake Meadows P.N.A., 45.8227°N, 66.1209°W, 13–25.V.2011, 2–21.VI.2011, 5–19.VII.2011, M. Roy & V. Webster, old silver maple forest and seasonally flooded marsh, Lindgren funnel traps (3, AFC, NBM, RWC). **Restigouche Co.**, Dionne Brook P.N.A., 47.9030°N, 68.3503°W, 27.VI-14.VII.2011, M. Roy & V. Webster, old-growth northern hardwood forest, Lindgren funnel trap (1, NBM). **Sunbury Co.**, Acadia Research Forest, 45.9866°N, 66.3841°W, 28.IV-4.V.2009, 25.V-2.VI.2009, R. Webster & M.-A. Giguère, mature (100 year-old) red spruce forest with scattered red maple and balsam fir, Lindgren funnel traps (1 ♂, 1 sex undetermined, AFC, RWC). **York Co.** New Maryland, off Hwy 2, E of Baker Brook, 45.8760°N, 66.6252°W, 6.IV.2005, R. P. Webster, old growth eastern white cedar swamp, in moss and litter at base of cedar (1 ♂, 1 ♀, RWC); 15 km W of Tracy, off Rt. 645, 45.6848°N, 66.8821°W, 4–11.V.2009, 11–19.V.2009, 28.VI-7.VII.2009, R. Webster & M.-A. Giguère, mature (120- 180 year-old) red pine forest, Lindgren funnel traps (1 ♀, 2 sex undetermined, AFC, RWC); Charters Settlement, 45.8395°N, 66.7391°W, 16–23.V.2009, R. P. Webster, mixed forest, Lindgren funnel trap (1 ♂, RWC).

####### Collection and habitat data.

Little is known about the habitat associations of this species. Smetana (1995) speculated that it might live in burrows of mammals or, less likely, in bird nests. Two specimens from New Brunswick were found in moss at the base of a tree in an old-growth eastern white cedar swamp early in the season when some snow and ice were still present. This was possibly an overwintering site. Most specimens were captured in Lindgren funnel traps deployed in a variety of forest types (red pine, red oak, red spruce, mixed forest, old-growth northern hardwood forest). These traps mimic tree trunks (Lindgren 1983), and it is possible that this species lives in microhabitats associated with standing trees. Adults were collected in April, May, June, and July.

####### Distribution in Canada and Alaska.

QC, **NB** (Smetana 1995). This rare species was known from only a few localities in Quebec south to Massachusetts and west to Michigan, Kansas, and South Dakota (Smetana 1995).

**Map 30.**
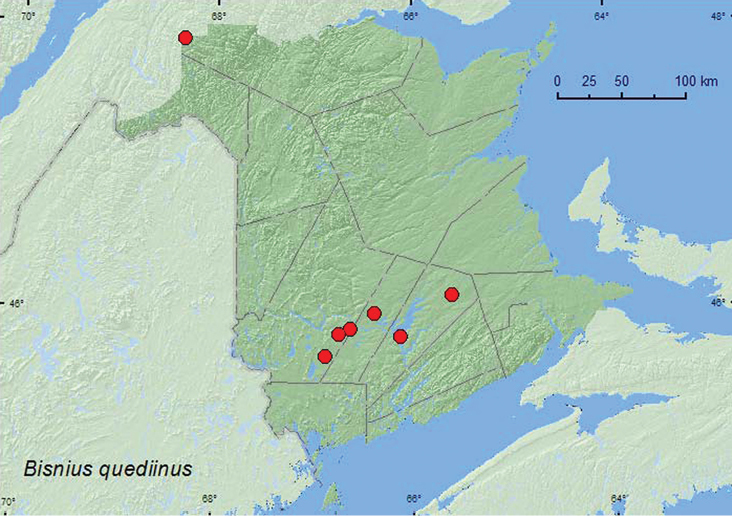
Collection localities in New Brunswick, Canada of *Bisnius quediinus*.

###### Erichsonius alumnus

Frank, 1975**

http://species-id.net/wiki/Erichsonius_alumnus

Map 31

####### Material examined.

**New Brunswick, Charlotte Co.**, ca. 9 km NW of New River, 45.2067°N, 66.6505°W, 13.VI.2008, R. P. Webster, alder swamp near large brook, treading vegetation along brook margin (1 ♀, NBM). **Restigouche Co.**, Little Tobique River near Red Brook, 47.4465°N, 67.0689°W, 13.VI.2006, R. P. Webster, river margin, under debris on clay and sand mix (3 ♂, 5 sex undetermined, RWC). **York Co.**, Charters Settlement, 45.8404°N, 66.7360°W, 27.V.2008, R. P. Webster, brook margin partially shaded by alders, among cobblestones and gravel (1 ♂, RWC); near Mazerolle Settlement, N of Hwy 2 near exit 271, 45.8764°N, 66.8260°W, 7.VI.2008, R. P. Webster, brook margin in beaver meadow, in fine gravel/clay mixed with grass (1 ♂, RWC); 8.4 km W of Tracy, off Rt. 645, 45.6821°N, 66.7894°W, 14.V.2008, R. P. Webster, alder swamp near brook, in leaf litter and grass on hummock (1, NBM).

####### Collection and habitat data.

*Erichsonius alumnu*s appears to be a riparian species associated with river and brook margins. Records in Frank (1975) included individuals from banks of streams, on mud by a brook, and an individual collected by treading mud by a stream. In New Brunswick, the largest series was collected along a river margin under debris on a sand–clay mix. Other adults were collected from brook margins in alder swamps and a beaver meadow. Adults occurred in grass litter mixed with fine gravel and clay, among cobblestones and gravel, and leaf and grass litter from these brook margin habitats. This species was collected in May and June.

####### Distribution in Canada and Alaska.

ON, QC, **NB** (Frank 1975).

**Map 31.**
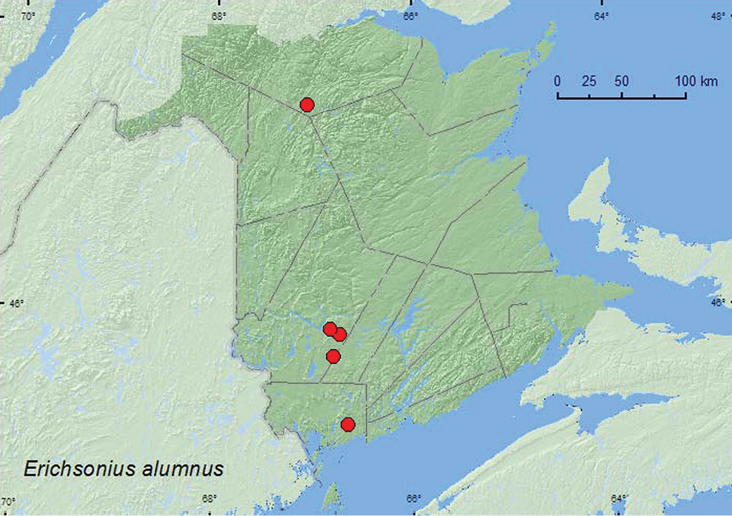
Collection localities in New Brunswick, Canada of *Erichsonius alumnus*.

###### Erichsonius inutilis

(Horn, 1884)**

http://species-id.net/wiki/Erichsonius_inutilis

Map 32

####### Material examined.

**New Brunswick, Queens Co.**, W of Jemseg at “Trout Creek”, 45.8231°N, 66.1245°W, 11.IV.2006, R. P. Webster, silver maple swamp, sifting leaf litter from crotch of silver maple with multiple trunks (1 ♂, 1 sex undetermined, RWC); near Queenstown, 45.6904°N, 66.1455°W, 13.V.2008, R. P. Webster, old hardwood forest, in leaf litter in seepage area near small brook (2 ♂, 1 sex undetermined, RWC); ca. 3.5 km W of Lower Gagetown, 45.7497°N, 66.1846°W, 13.V.2008, R. P. Webster, mature red oak and red maple forest, in moist leaves on margin of vernal pond (1, RWC). **York Co.**, Mazerolle Settlement, 45.8729°N, 66.8311°W, 28.IV.2006, R. P. Webster, stream margin (sun-exposed), in grass litter on muddy soil (1 ♂, RWC); Kelly’s Creek at Sears Road, 45.8723°N, 66.8414°W, 7.VI.2008, R. P. Webster, *Carex* marsh, treading (1 ♂, RWC); Fredericton at Saint John River, 45.9598°N, 66.6258°W, 19.VII.2005, R. P. Webster, river margin, under drift material (1, RWC).

####### Collection and habitat data.

Little was previously known about the habitat associations of this species. The only records with habitat data reported in Frank (1975) included specimens sifted from flood debris, swamp grass, ex nest of a meadow vole (*Microtus pennsylvanicus* Ord), and one individual collected in a tamarack bog. In New Brunswick, this species appears to be associated with moist to wet habitats in forested areas, including seepage areas near small brooks, vernal pond margins, stream and river margins, and *Carex* marsh. Adults were sifted from moist leaves, grass litter on muddy soil, drift material, and by treading *Carex*. A few adults were sifted from leaf litter from the crotch of a silver maple with multiple trunks in early April in a silver maple swamp. This was presumably an overwintering site for these adults. Adults were collected during April, May, June, and July.

####### Distribution in Canada and Alaska.

ON, QC, **NB** (Frank 1975).

**Map 32.**
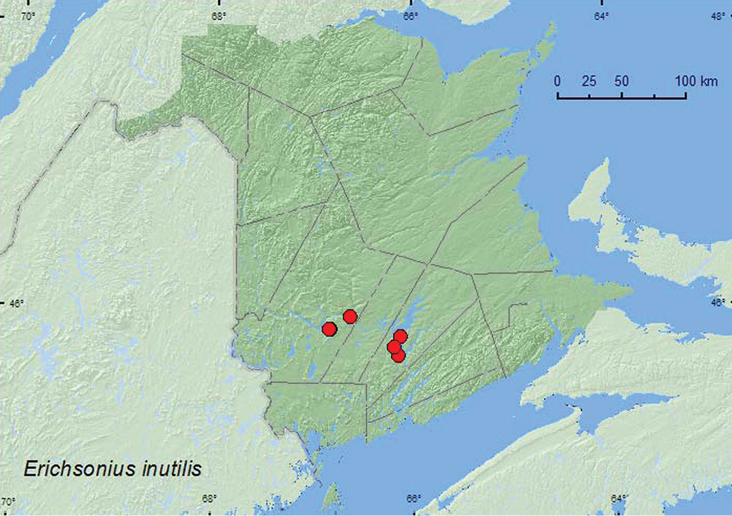
Collection localities in New Brunswick, Canada of *Erichsonius inutilis*.

###### Erichsonius parcus

(Horn, 1884)**

http://species-id.net/wiki/Erichsonius_parcus

Map 33

####### Material examined.

**New Brunswick, York Co.**, New Maryland, U.N.B. Woodlot, 45.9116°N, 66.6698°W, 26.V.2008, R. Webster, G. Forbes, & M.-A. Giguère, abandoned beaver lodge occupied by muskrats, in wall of lodge (4 ♂, 2 ♀, RWC).

####### Collection and habitat data. 

Little was previously known about the habitat associations of *Erichsonius parcus*. The specimens from New Brunswick were collected from the wall of an abandoned beaver lodge occupied by muskrats. In Rhode Island (USA) (Washington Co., 2 mi S of Shannock, 41.4270°N, 71.6039°W, 22.IV.2007), three specimens were sifted from moist leaves on the margin of a vernal pond (Webster, unpublished data) in a red maple swamp.

####### Distribution in Canada and Alaska.

ON, **NB** (Brunke and Marshall 2011). *Erichsonius parcus* was reported from Massachusetts, south to Virginia, South Carolina, and Louisiana in the USA by Frank (1975).

**Map 33.**
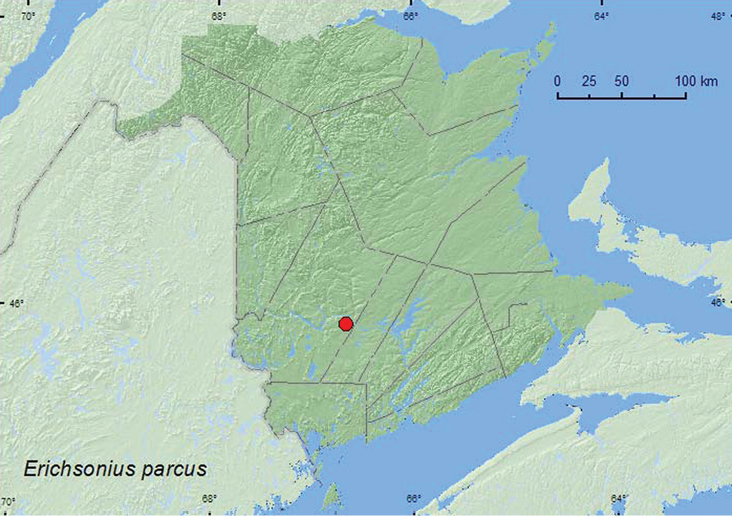
Collection localities in New Brunswick, Canada of *Erichsonius parcus*.

###### Erichsonius patella

(Horn, 1884)

http://species-id.net/wiki/Erichsonius_patella

Map 34

####### Material examined.

**New Brunswick, Queens Co.**, Grand Lake near Scotchtown, 45.8762°N, 66.1816°W, 25.V.2006, R. P. Webster, lakeshore, in drift material (1 ♂, RWC); Rees, near Grand Lake, 46.0016°N, 65.9466°W, 29.V.2007, S. Makepeace & R. Webster, coll., nest box contents of barred owl (1 ♀, NBMB). **Sunbury Co.**, Acadia Research Forest, 45.9866°N, 66.3841°W, 19–25.V.2009, R. Webster & M.-A. Giguère, mature (110 year-old) red spruce forest with scattered red maple and balsam fir, Lindgren funnel trap (1 ♀, AFC). **York Co.**, Charters Settlement, 45.8340°N, 66.7450°W, 15.V.2004, 30.V.2004, 10.VI.2004, 27.IV.2005, R. P. Webster, mixed forest, under conifer bark in wood pile (4 sex undetermined, RWC); same locality and collector but 45.8300°N, 66.7360°W, 20.VI.2004, R. P. Webster, mature mixed forest, in leaf litter near stream (1, RWC); same locality and collector but 45.8395°N, 66.7391°W, 28.IX.2005, mixed forest, in compost (decaying vegetables) (1 ♂, RWC); Fredericton, Odell Park, 45.9570°N, 66.6695°W, 19.VI.2005, R. P. Webster, in pile of woodchips and decaying plant materials (1 ♂, 1 sex undetermined, RWC); Nashwaaksis River at Rt. 105, 45.9850°N, 66.6900°W, 6.V.2006, R. P. Webster, upper river margin in flood debris (1, RWC); Kelly’s Creek at Sears Road, 45.8723°N, 66.8414°W, 7.VI.2008, R. P. Webster, alder swamp with red maple, in moist leaf and grass litter near vernal pool (1 sex undetermined, NBM); 15 km W of Tracy, off Rt. 645, 45.6848°N, 66.8821°W, 18.V-2.VI.2010, R. Webster & C. MacKay, mature (120–180 year-old) red pine forest, Lindgren funnel trap (1, AFC).

####### Collection and habitat data.

This species appears to be associated with various kinds of decaying organic materials. The few records with habitat data reported in Frank (1975) included adults collected from human dung, sifting leaves, sifting oak leaves, in ground cover in a white pine forest, ex mushrooms, funnel extract of mixed litter, *Rhododendron* litter, and pine–hardwood leaf litter near a stream edge. In New Brunswick, adults were found in a variety of decaying organic material, including drift material along a lakeshore, flood debris on an upper river margin, leaf litter near a stream, moist leaf and grass litter near a vernal pool, under conifer bark in a wood pile, in compost (decaying vegetables), in a pile of woodchips, and on decaying plant material. A few individuals were captured in Lindgren funnel traps. Most collections were from forested habitats. One adult was collected from the nest contents of a barred owl. Adults were collected in April, May, and June.

####### Distribution in Canada and Alaska.

ON, QC, **NB**, NS (Frank 1975; Bishop et al. 2009).

**Map 34.**
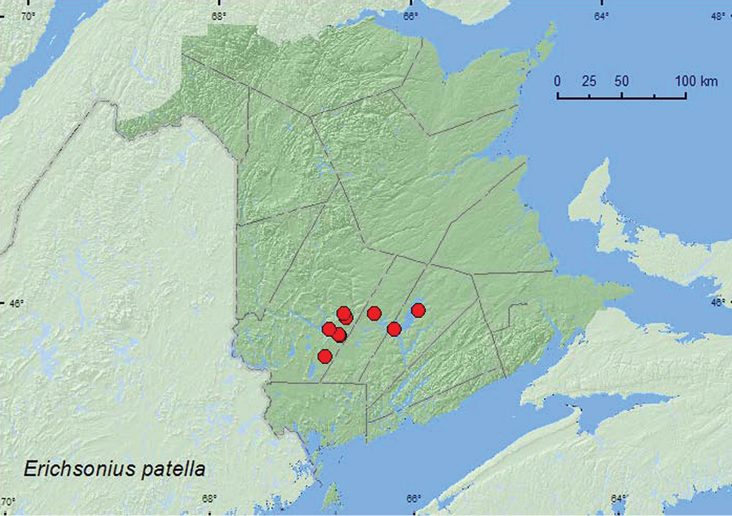
Collection localities in New Brunswick, Canada of *Erichsonius patella*.

###### Erichsonius pusio

(Horn, 1884)**

http://species-id.net/wiki/Erichsonius_pusio

Map 35

####### Material examined.

**New Brunswick, York Co.**, Mazerolle Settlement, 45.8729°N, 66.8311°W, 28.IV.2006, R. P. Webster, stream margin (sun exposed), in grass litter on muddy soil (1 ♂, 1 ♀, 2 sex undetermined, RWC); near Mazerolle Settlement, N of Hwy 2 near exit 271, 45.8764°N, 66.8260°W, 7.VI.2008, R. P. Webster, brook margin in beaver meadow, in fine gravel/clay mixed with grass (2 ♂, 2 sex undetermined, RWC); Charters Settlement, 45.8395°N, 66.7391°W, 6.V.2008, R. P. Webster, mixed forest, in flight, collected with aerial net between 15:00 and 17:00 h (1 ♂, RWC).

####### Collection and habitat data.

Little was previously known about the habitat associations of this species. The only records with habitat data reported in Frank (1975) included a specimen collected from a funnel extract of oak–beech leaf litter and an individual in a windrow sample. In New Brunswick, most specimens were sifted from grass litter on muddy soil or from litter with grass mixed with fine gravel/clay along the margins of a stream through a beaver meadow. One adult was collected with an aerial net during evening flight. Adults were collected in April, May, and June.

####### Distribution in Canada and Alaska.

BC, ON, QC, **NB** (Frank 1975).

**Map 35.**
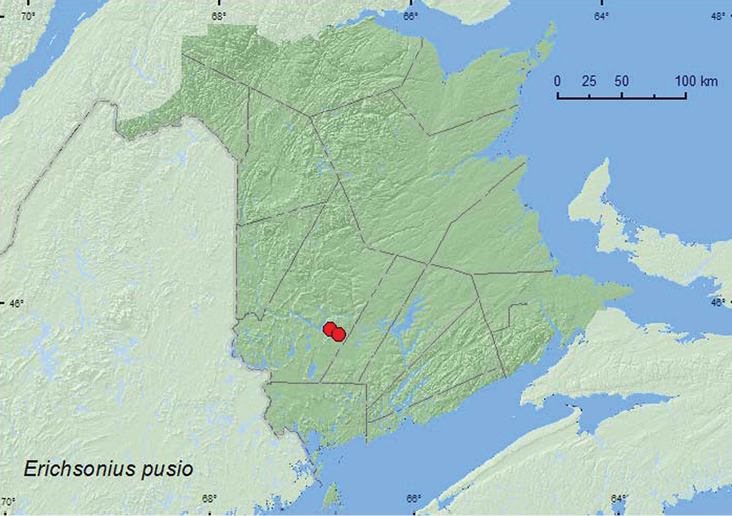
Collection localities in New Brunswick, Canada of *Erichsonius pusio*.

###### Erichsonius rosellus

Frank, 1975**

http://species-id.net/wiki/Erichsonius_rosellus

Map 36

####### Material examined.

**New Brunswick, Charlotte Co.**, near Clark Ridge, 45.3155°N, 67.4406°W, 27.V.2007, R. P. Webster, beaver pond, treading vegetation (1, RWC). **Restigouche Co.**, Jacquet River Gorge P.N.A., 47.7357°N, 66.0774°W, 24.VI.2008, R. P. Webster, beaver pond margin, among leaves and sedges (1 ♂, RWC). **Sunbury Co.**, Maugerville, Portobello Creek N.W.A., 45.8992°N, 66.4248°W, 5.VI.2004, R. P. Webster, silver maple swamp, in leaf litter on margin of small pond (1 ♂, RWC). **York Co.** 8.5 km W of Tracy, off Rt. 645, 45.6821°N, 66.7894°W, 6.V.2008, R. P. Webster, wet alder swamp in leaf litter and grass on hummock (1 ♂, RWC); Fredericton, University of New Brunswick Woodlot, 45.9116°N, 66.6698°W, 26.V.2008, R. Webster, G. Forbes, & M.-A. Giguère, abandoned beaver lodge occupied by muskrats, in roof of lodge (3 ♂, 1 sex undetermined, RWC); University of New Brunswick Woodlot, 45.9391°N, 66.6747°W, 17.VIII.2009, R. Webster, D. McAlpine, & G. Forbes, in beaver lodge, within wall of lodge (1 ♂, RWC).

####### Collection and habitat data.

Almost nothing was previously known about the habitat associations of this species. The only record with habitat data reported in Frank (1975) included a specimen collected from a “pool seep”. In New Brunswick, adults were found in leaf litter in wet habitats, such as beaver pond margins, a pond margin in a silver maple swamp, and a wet alder swamp. This species was also found in the walls of both a beaver lodge and an abandoned beaver lodge occupied by muskrats. Adults were collected in May, June, and August.

####### Distribution in Canada and Alaska.

ON, QC, **NB** (Frank 1975).

**Map 36.**
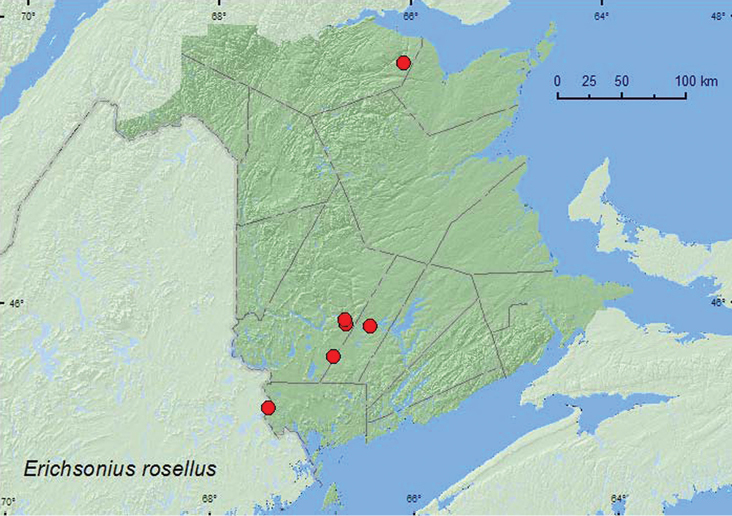
Collection localities in New Brunswick, Canada of *Erichsonius rosellus*.

###### Gabrius appendiculatus

Sharp, 1910

http://species-id.net/wiki/Gabrius_appendiculatus

Map 37

####### Material examined.

**Additional New Brunswick records, Carleton Co.**, Meduxnekeag River Valley Nature Preserve, 46.1931°N, 67.6825°W, 15.IX.2004, R. P. Webster, river margin, under drift material (1 sex undetermined, RWC); Two Mile Brook Fen, 46.3619°N, 67.6733°W, 6.V.2005, M.-A. Giguère & R. Webster, eastern white cedar swamp, in moist sphagnum (1, NBM). **Queens Co.**, Grand Lake near Scotchtown, 45.8762°N, 66.1816°W, 12.V.2004, R. P. Webster, lakeshore, under drift material (1 ♂, RWC); Bayard (near Welsford) near Nerepis River, 45.4442°N, 66.3292°W, 25.V.2008, R. P. Webster, pond margin, in moist grass litter on mud (1 ♂, RWC). **Sunbury Co.**, Acadia Research Forest, 45.9816°N, 66.3374°W, 18.VII.2007, R. P. Webster, regenerating mixed forest (8.5 years-old), in sphagnum and leaf litter at bottom of dried vernal pool (1, RWC). **York Co.**, Charters Settlement, 45.8395°N, 66.7391°W, 22.IV.2004, 9.IV.2005, R. P. Webster, residential lawn in grass (1 ♂, 1 sex undetermined, RWC); same locality, forest type, and collector, 17.IV.2005, in flight, collected with aerial net during warm afternoon (1 sex undetermined, RWC); same locality and collector, 29.III.2006, mixed forest under alders near brook, in leaf litter (1 ♂, RWC); Fredericton, at Saint John River, 45.9588°N, 66.6254°W, 7.VI.2005, R. P. Webster, river margin, in flood debris (1 ♂, RWC); Keswick River at Rt. 105, 45.9938°N, 66.8344°W, 3.VI.2008, R. P. Webster, silver maple swamp near river margin, in leaf and grass litter on mud/clay soil (1 ♂, RWC); 14 km WSW of Tracy, S of Rt. 645, 45.6741°N, 66.8661°W, 25.IV-4.V.2010, R. Webster & C. MacKay, old mixed forest with red and white spruce, red and white pine, balsam fir, eastern white cedar, red maple, and *Populus* sp., Lindgren funnel trap (1, AFC).

####### Collection and habitat data.

In Europe, adults of this adventive species occur in wet habitats, marshes, swamps, margins of streams and ponds, and swampy meadows; it is found in similar habitats in North America (Smetana 1995). In New Brunswick, most adults were found along lake, pond, brook, and river margins, usually in leaf litter or drift material. Others were found in an eastern white cedar swamp in moist sphagnum, in a dried vernal pond in sphagnum and leaf litter. Klimaszewski et al. (2005) reported specimens from pitfall traps deployed in a red spruce stand (Acadia Research Forest). Adults were collected in March, April, May, June, July, and September.

####### Distribution in Canada and Alaska.

BC, ON, QC, **NB**, NF (Smetana 1995; Klimaszewski et al. 2005; Brunke and Marshall 2011). *Gabrius appendiculatus*was first reported from New Brunswick by Klimaszewski et al. (2005).

**Map 37.**
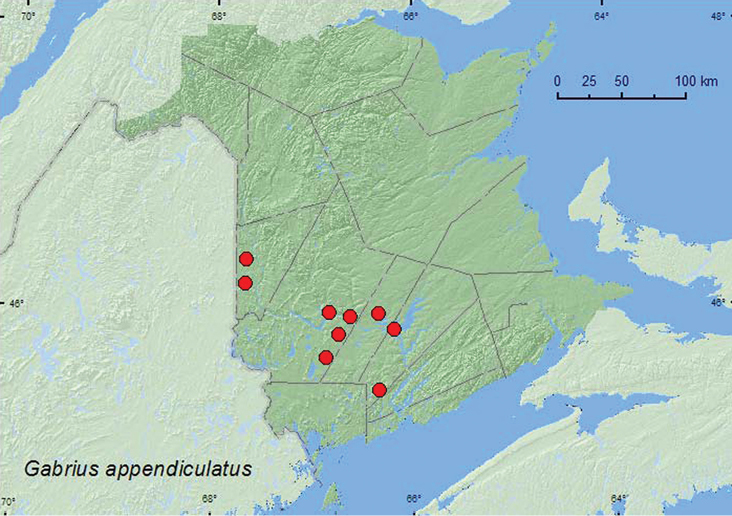
Collection localities in New Brunswick, Canada of *Gabrius appendiculatus*.

###### Gabrius fallaciosus

(Horn 1884)**

http://species-id.net/wiki/Gabrius_fallaciosus

Map 38

####### Material examined.

**New Brunswick, Carleton Co.**, Jackson Falls, Bell Forest, 46.2152°N, 67.7190°W, 1.VI.2005, M.-A. Giguère & R. Webster, upper river margin, in flight, collected with aerial net between 16:00 and 18:00 h (1, RWC); Hovey Hill P.N.A., 46.1115°N, 67.7770°W, 10.V.2005, 24.V.2005, R. P. Webster, hardwood forest, under bark of *Fagus grandifolia* (American beech) (1 ♂, 1 sex undetermined, RWC); Meduxnekeag Valley Nature Preserve, 46.1883°N, 67.6745°W, 9.VIII.2005, M.-A. Giguère & R. Webster, old hardwood forest, under bark (1 ♂, RWC). **Charlotte Co.**, 3.0 km NW of Pomeroy Ridge, 45.3059°N, 67.4343°W, 5.VI.2008, R. P. Webster, red maple and eastern white cedar swamp, under bark of red maple (1 ♂, NBM); 10 km NW of New River Beach, 45.2110°N, 66.6170°W, 30.IV-17.V.2010, R. Webster & V. Webster, coll., old growth eastern white cedar forest, Lindgren funnel trap (1, AFC). **Queens Co.**, ca. 3.5 km W of Lower Gagetown, 45.7497°N, 66.1846°W, 13.V.2008, R. P. Webster, mature red oak and red maple forest, under bark (red oak) (1 ♂, RWC); Central Hampstead, 45.6437°N, 66.1462°W, 13.V.2008, R. P. Webster, mixed forest, under bark of hardwood (1, RWC);Cranberry Lake P.N.A., 46.1125°N, 65.6075°W, 24.IV-5 V.2009, 21–27.V.2009, R. Webster & M.-A. Giguère, mature red oak forest, Lindgren funnel traps (4, AFC). **Sunbury Co.**, Noonan, 45.9923°N, 66.4099°W, 2.VI.2007, S. Makepeace & R. Webster, coll., nest contents of barred owl from tree hole 7 m high in red maple (1, RWC). **York Co.,** Charters Settlement, 45.8395°N, 66.7391°W, 1.V.2005, R. P. Webster, mixed forest, under bark of conifer log (2, RWC); same locality and collector but 45.8286°N, 66.7365°W, 2.VI.2007, old red spruce and red maple forest, under scolytid-infested bark of red spruce (1 ♂, RWC); 14 km WSW of Tracy, S of Rt. 645, 45.6741°N, 66.8661°W, 26.IV-10.V.2010, R. Webster & C. MacKay, coll., old mixed forest with red and white spruce, red and white pine, balsam fir, eastern white cedar, red maple, and *Populus* sp., Lindgren funnel trap (1, AFC).

####### Collection and habitat data.

Smetana (1995) reported that this species typically occured under bark of both deciduous and coniferous species and was often associated with old trees. Adults occur in debris under bark, in rotting wood, and in wood and debris around the bases of dead standing trees. This species has also been found in rotting mushrooms and forest floor litter and has been taken in flight intercept traps (Smetana 1995). In New Brunswick, this species was found in similar habitats, including under bark of American beech, red maple, red oak, red spruce, and from the nest contents of a barred owl. Adults were also collected in Lindgren funnel traps and in flight during evening flights. Adults were captured during April, May, June, and August.

####### Distribution in Canada and Alaska.

ON, QC, **NB** (Smetana 1995).

**Map 38.**
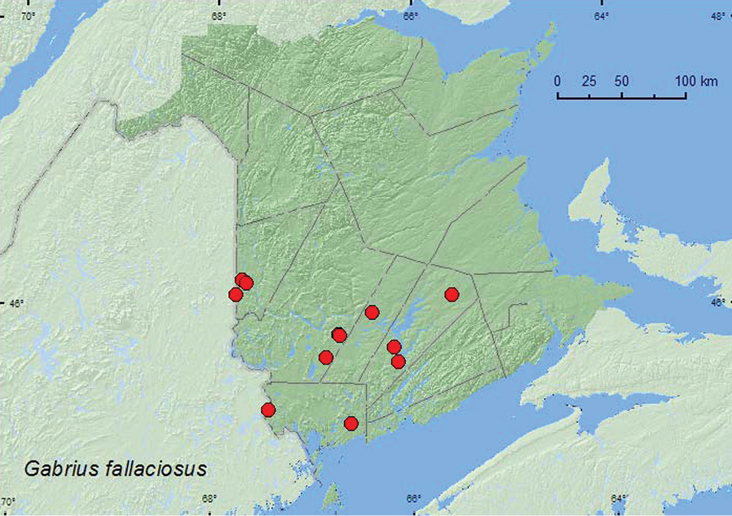
Collection localities in New Brunswick, Canada of *Gabrius fallaciosus*.

###### Gabrius ulpius

Smetana, 1995

http://species-id.net/wiki/Gabrius_ulpius

Map 39

####### Material examined.

**New Brunswick, Restigouche Co.**, Little Tobique River near Red Brook, 47.4462°N, 67.0689°W, 24.V.2007, R. P. Webster, old growth eastern white cedar forest, in moss and leaf litter near brook (1 ♂, RWC).

####### Collection and habitat data.

This species has been collected most frequently from deciduous forest habitats and has been collected from forest floor debris (moist deep leaf litter layers), in moss on rocks, in old mushrooms, and in rotten wood (Smetana 1995). The single New Brunswick specimen was sifted from moss and leaf litter adjacent to a brook in an old-growth eastern white cedar forest.

####### Distribution in Canada and Alaska.

ON, QC, **NB**, NS (Smetana 1995). It is apparent from the above record that *Gabrius ulpius* is probably more continuously distributed in the Maritime provinces than was suggested by Majka et al. (2009).

**Map 39.**
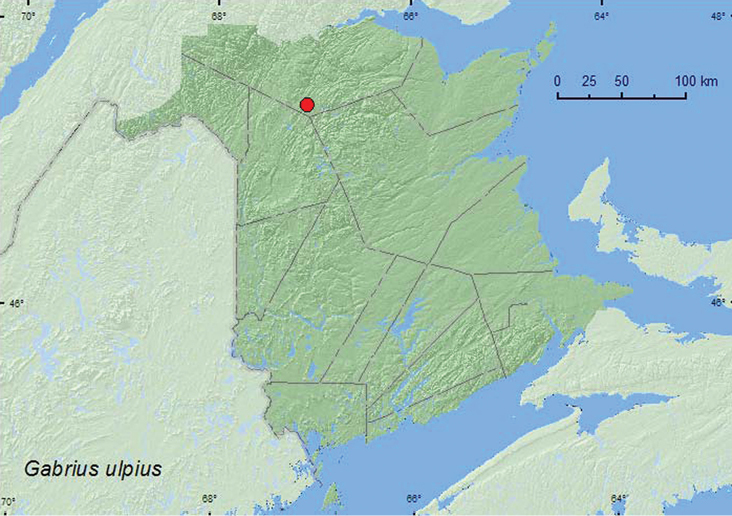
Collection localities in New Brunswick, Canada of *Gabrius ulpius*.

###### Hesperus apicialis

(Say, 1830)**

http://species-id.net/wiki/Hesperus_apicialis

Map 40

####### Material examined.

**New Brunswick, Queens Co.**, Cranberry Lake P.N.A., 46.1125°N, 65.6075°W, R. Webster & M.-A. Giguère, 24.IV.-5.V.2009, red oak forest, Lindgren funnel trap (1 ♂, RWC); same locality data and forest type, 29.VI-7.VII.2011, 7–13.VII.2011, M. Roy & V. Webster, Lindgren funnel traps in forest canopy (2, NBM, RWC). **Sunbury Co.**, Noonan, 45.9923°N, 66.4099°W, 2.VI.2007, S. Makepeace & R. Webster, coll., nest contents (damp organic material with small bones) of barred owl from tree hole 7 m high in red maple, (1 ♀, RWC); Acadia Research Forest, 45.9866°N, 66.3841°W, 9–16.VI.2009, R. Webster & M.-A. Giguère, red spruce forest (100 years old) with red maple and balsam fir, Lindgren funnel trap (1 ♀, RWC).

####### Collection and habitat data.

*Hesperus apicialis*is associated with old trees, occurring under bark and in wood and debris of old, fallen, deciduous and coniferous trees and in tree holes (Smetana 1995). In New Brunswick, one adult was collected from the nest contents of a barred owl nesting in a tree hole. Other adults were collected from Lindgren funnel traps in red oak and red spruce forests. Two adults were captured in Lindgren funnel traps deployed in the forest canopy. Adults were collected in April, May, June, and July.

####### Distribution in Canada and Alaska.

ON, QC, **NB** (Smetana 1995).

**Map 40.**
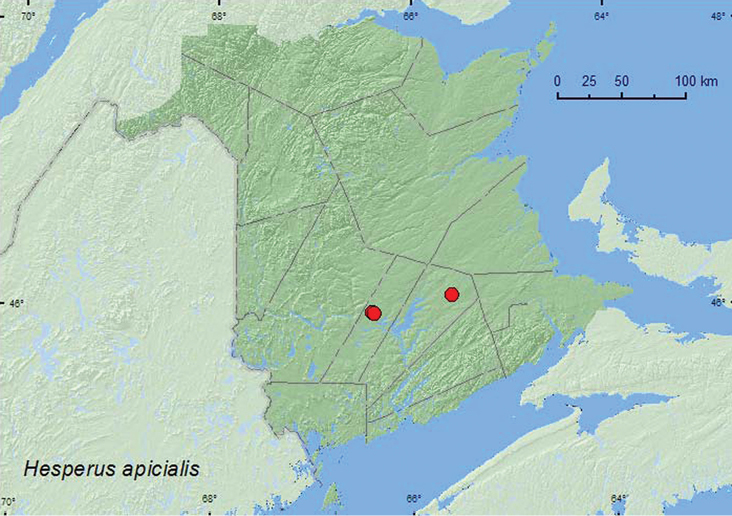
Collection localities in New Brunswick, Canada of *Hesperus apicialis*.

###### Laetulonthus laetulus

(Say, 1834)**

http://species-id.net/wiki/Laetulonthus_laetulus

Map 41

####### Material examined.

**New Brunswick, Carleton Co.**, Jackson Falls, Bell Forest, 46.2200°N, 67.7231°W, 12–19.VI.2008, R. P. Webster, mature hardwood forest, Lindgren funnel trap (1 ♂, RWC); same locality data and collector, 12.IX.2008, in fleshy polypore mushroom on beech log (1, RWC). **Charlotte Co.**, 10 km NW of New River Beach, 45.2110°N, 66.6170°W, 31.V-15.VI.2010, R. P. Webster and V. Webster, coll., old-growth eastern white cedar forest, Lindgren funnel trap (1, AFC). **Queens Co.**, Cranberry Lake P.N.A., 46.1125°N, 65.6075°W, 4–18.VIII.2011, M. Roy & V. Webster, old red oak forest, Lindgren funnel trap (1, NBM). **York Co.**, Charters Settlement, 45.8340°N, 66.7450°W, 14.V.2004, 30.V.2004, R. P. Webster, mixed forest, in wood pile, under bark of spruce log (1 ♂, 1 ♀, NBM, RWC); same locality and collector, 45.8395°N, 66.7391°W, 16.X.2004, 28.IX.2005, 22.VIII.2005, 8.VIII.2008, mixed forest, in plastic compost bin with decaying vegetables (3 ♂, 3 ♀, RWC); 15 km W of Tracy off Rt. 645, 45.6848°N, 66.8821°W, 15–21.VI.2009, 29.VII-4 VIII.2009, 4–11.VIII.2009, R. Webster & M.-A. Giguère, Lindgren funnel traps (6, AFC); 14 km WSW of Tracy, S of Rt. 645, 45.6741°N, 66.8661°W, 10–26.V.2010, 26.V-2.VI.2010, R. Webster & C. MacKay, coll., old mixed forest with red and white spruce, red and white pine, balsam fir, eastern white cedar, red maple, and *Populus* sp., Lindgren funnel traps (2, AFC).

####### Collection and habitat data.

This species is associated with deciduous and coniferous trees, usually occurring in debris around bases of trees, in tree holes, under bark, and in rotting wood of old fallen trees (Smetana 1995). In New Brunswick, specimens were collected from compost (decaying vegetables) in a compost bin, from under bark of a spruce log, in a fleshy polypore mushroom on a beech log, and from Lindgren funnel traps. Adults were collected in May, June, July, August, and September.

####### Distribution in Canada and Alaska.

ON, QC, **NB** (Smetana 1995).

**Map 41.**
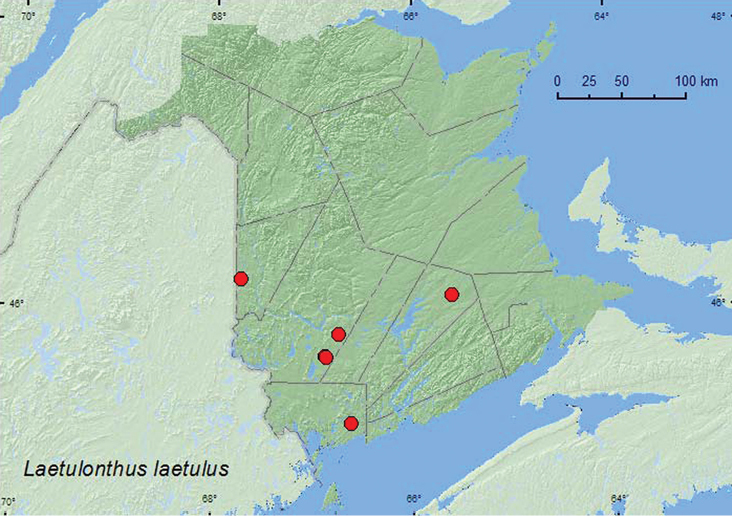
Collection localities in New Brunswick, Canada of *Laetulonthus laetulus*.

###### Neobisnius jucundus

(Horn, 1884)**

http://species-id.net/wiki/Neobisnius_jucundus

Map 42

####### Material examined.

**New Brunswick, Queens Co.**, Grand Lake, on Goat Island, 46.0110°N, 66.0133°W, 17.VIII.2004, D. Sabine & R. Webster, lake margin among cobblestones in grassy area near shoreline (1 ♂, 2 sex undetermined, RWC); Grand Lake at Cox Point, 46.0161°N, 65.9942°W, 17.VIII.2004, D. Sabine & R. Webster, lake margin among cobblestones near shoreline (3, RWC).

####### Collection and habitat data.

Little is known about the habitat associations of this species. The few records with habitat data reported in Frank (1981) included salt marsh margins, a sand bar, and under drift on a (sea) beach. Some other collection localities were near lakes and river systems. All the New Brunswick specimens were found under cobblestones near the margin of a lake during August.

####### Distribution in Canada and Alaska.

BC, AB, ON, **NB** (Frank 1981).

**Map 42.**
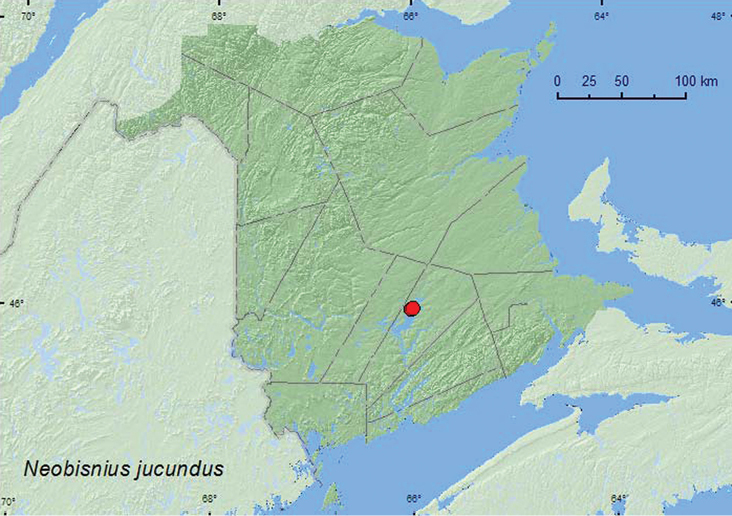
Collection localities in New Brunswick, Canada of *Neobisnius jucundus*.

###### Neobisnius lathrobioides

(Baudi, 1848)**

http://species-id.net/wiki/Neobisnius_lathrobioides

Map 43

####### Material examined.

**New Brunswick, Restigouche Co.**, Jacquet River Gorge P.N.A., 47.8257°N, 66.0779°W, 24.V.2010, R. P. Webster, partially shaded cobblestone and sand bar near outflow of brook at the Jacquet River, under cobblestone on sand (1 ♂, RWC).

####### Collection and habitat data.

No bionomic information was reported in Frank (1981) for this adventive species. The single New Brunswick specimen was under a small rock on a cobblestone bar near a brook and river. The adult was collected in May.

####### Distribution in Canada and Alaska.

QC, **NB** (Frank 1981).

**Map 43.**
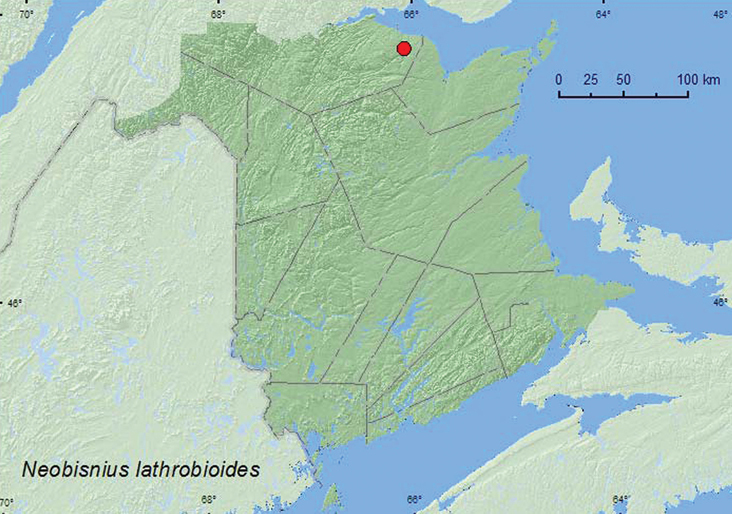
Collection localities in New Brunswick, Canada of *Neobisnius lathrobioides*.

###### Neobisnius terminalis

(LeConte, 1863)

http://species-id.net/wiki/Neobisnius_terminalis

Map 44

####### Material examined.

**Additional New Brunswick records, Carleton Co.**, Hartland, (old) Hwy 2 at Saint John River, 46.3136°N, 67.5376°W, 2.VIII.2004, R. P. Webster, river margin, on moist clay among tall grasses (1 sex undetermined, RWC); Hartland, Becaguimec Island (in Saint John River), 46.3073°N, 67.5376°W, 23.VI.2006, R. Capozi & R. Webster, river margin among cobblestones near water (1 ♂, 2 sex undetermined, RWC); Hartland, Middle Becaguimec Island (in Saint John River), 46.3028°N, 67.5333°W, 23.VI.2006, R. Capozi & R. Webster, river margin among cobblestones near water (1 ♂, 2 sex undetermined, RWC); Meduxnekeag River Valley Nature Preserve, 46.1942°N, 67.6832°W, 9.VI.2008, R. P. Webster, river margin, under small cobblestone set in sand and fine gravel near water (1, RWC). **Madawaska Co.**, at Green River, 47.6918°N, 68.3202°W, 21.VI.2010, M. Turgeon & R. Webster, river margin, among gravel on gravel bar (1, NBM). **Northumberland Co.**, Amostown, at Miramichi River, 46.5339°N, 66.2094°W, 11.VIII.2006, R. P. Webster, river margin, among cobblestones near water (1, RWC). **York Co.**, 1.5 km N of Durham Bridge at Nashwaak River, 46.1408°N, 66.6179°W, 15.VI.2008, R. P. Webster, river margin, among cobblestones near outflow of brook (1 ♂, RWC).

####### Collection and habitat data.

Little is known about the habitat associations of this species. Most of the specimens collected in New Brunswick were found under cobblestones along river margins, usually close to water. Adults were collected in June and August.

####### Distribution in Canada and Alaska.

QC, NB, NS (Frank 1981). *Neobisnius terminalis* was listed as occurring in New Brunswick by Majka et al. (2011) without any supporting references or data. Here, we provide the first documented records from New Brunswick.

**Map 44.**
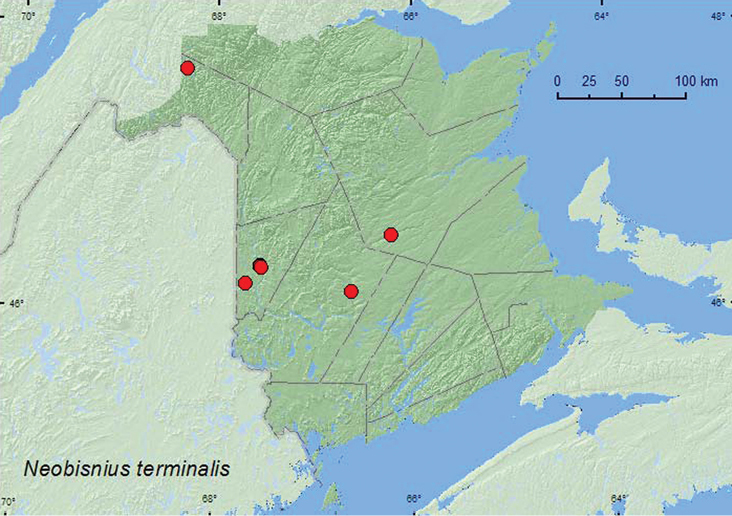
Collection localities in New Brunswick, Canada of *Neobisnius terminalis*.

###### Philonthus aequalis

Horn, 1884**

http://species-id.net/wiki/Philonthus_aequalis

Map 45

####### Material examined.

**New Brunswick, Carleton Co.**, Meduxnekeag Valley Nature Preserve, 46.1976°N, 67.6850°W, 4.V.2006, R. P. Webster, mixed forest, margin of vernal pond in leaf litter (1 ♂, RWC); same locality but 46.1942°N, 67.6832°W, 9.VI.2008, R. P. Webster, river margin, under cobblestone set in sand and fine gravel near water’s edge (1 ♂, RWC); Jackson Falls, Bell Forest, 46.2150°N, 67.7201°W, 14.V.2006, R. P. Webster, river margin, in drift material near seepage area (1 ♂, 1 ♀, RWC). **York Co.**, Fredericton, at Saint John River, 45.9588°N, 66.6254°W, 4.VIII.2004, R. P. Webster, river margin, in drift material (mostly silver maple seeds) (3 ♂, RWC).

####### Collection and habitat data.

*Philonthus aequalis*occurs in wet habitats along margins of rivers and creeks, in swamps, marshes, and lake margins. Adults occur in flood debris and can also be collected by treading and sifting leaf litter in swampy forests (Smetana 1995). Most adults from New Brunswick were found along river margins in drift material. One individual was collected from leaf litter on the margin of a vernal pond in a mixed forest. Adults were collected in May, June, and August.

####### Distribution in Canada and Alaska.

MB, ON, QC, **NB** (Smetana 1995).

**Map 45.**
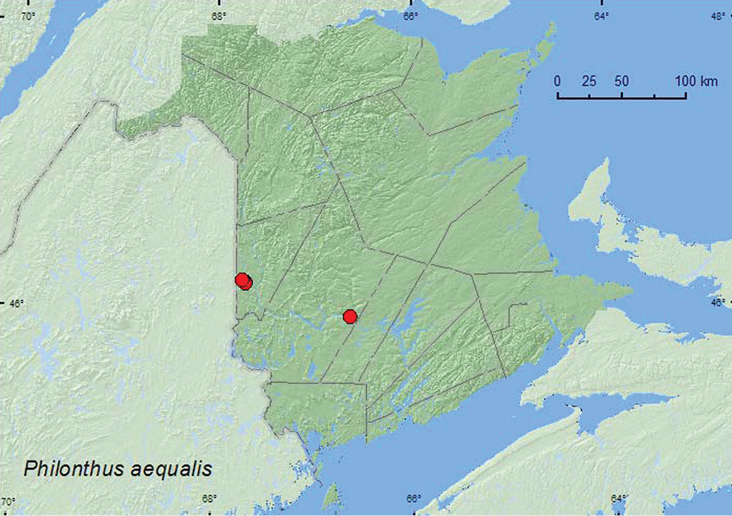
Collection localities in New Brunswick, Canada of *Philonthus aequalis*.

###### Philonthus boreas

Smetana, 1995**

http://species-id.net/wiki/Philonthus_boreas

Map 46

####### Material examined.

**New Brunswick, Queens Co.**, Grand Lake near Scotchtown, 45.8762°N, 66.1816°W, R. P. Webster, 12.V.2004, 25.V.2006, lakeshore, under drift material (1 ♂, 1 ♀, RWC).

####### Collection and habitat data.

*Philonthus boreas* has been collected from various wet habitats, including the muddy banks of a river among scattered low grassy vegetation, and by treading moss and grassy vegetation along lake and pond margins (Smetana 1995). In New Brunswick, adults of this northern transcontinental species were collected in drift material along a lakeshore in May.

####### Distribution in Canada and Alaska.

AK, YT, NT, BC, AB, SK, MB, ON, **NB**, NF (Smetana 1995).

**Map 46.**
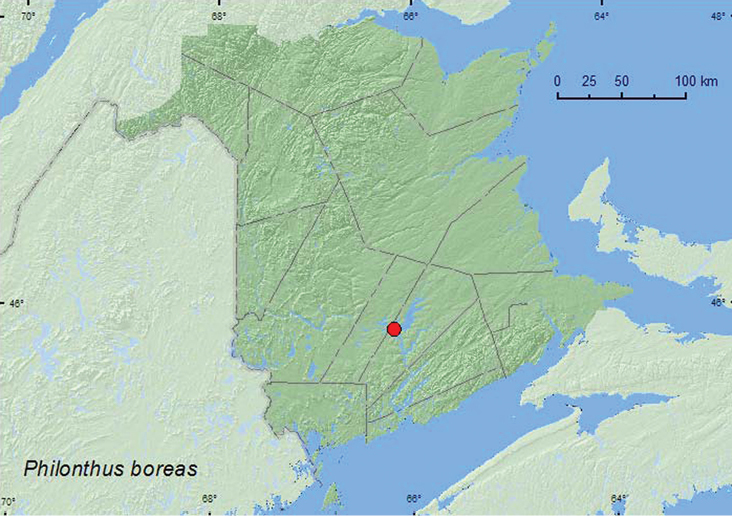
Collection localities in New Brunswick, Canada of *Philonthus boreas*.

###### Philonthus flavibasis

Casey, 1915**

http://species-id.net/wiki/Philonthus_flavibasis

Map 47

####### Material examined.

**New Brunswick, Carleton Co.**, “Two Mile Brook Fen”, 46.3619°N, 67.6733°W, 6.V.2005, M.-A. Giguère & R. Webster, eastern white cedar forest/swamp, in moist sphagnum (1 ♂, 2 ♀, NBM, RWC). **Charlotte Co.**, Rt. 3 at Deadwater Brook, 45.4744°N, 67.1225°W, 3.VI.2005, R. P. Webster, forested black spruce bog, in moist sphagnum (1 ♂, RWC); S of Little Pocologan River, 45.1537°N, 66.6269°W, 7.V.2007, R. P. Webster, black spruce and tamarack bog, in moss and litter (2 ♀, NBM, RWC); 3.5 km NW of Pomeroy Ridge, 45.3087°N, 67.4362°W, 5.V.2008, 18.VI.2008, R. P. Webster, red maple swamp, in sphagnum with grasses near margin of vernal pool (6 ♂, 1 ♀, NBM, RWC). **Northumberland Co.,** Goodfellow Brook P.N.A., 46.8943°N, 65.3796°W, 23.V.2007, R. P. Webster, old-growth eastern white cedar swamp, in grass litter and moss on hummocks near pool (1 ♂, NBM). **Restigouche Co.**, NE of jct. Little Tobique River and Red Brook, 47.4501°N, 67.0577°W, 24.V.2007, R. P. Webster, old growth eastern white cedar swamp, in moist sphagnum (2 ♂, 1 ♀, NBM, RWC); Jacquet River Gorge P.N.A., 47.8199°N, 66.0010°W, 25.VI.2008, R. P. Webster, mixed forest, margin vernal pool among moist leaves (1 ♂, NBM). **Sunbury Co.**, Acadia Research Forest, 46.0173°N, 66.3741°W, 18.IX.2007, R. P. Webster, 8.5 year-old regenerating mixed forest, in sphagnum and leaf litter in old tire depression (4 ♂, 1 ♀, NBM, RWC). **York Co.**, trail to Browns Mtn. Fen, 45.8978°N, 67.6273°W, 2.V.2005, M.-A. Giguère & R. Webster, mature eastern white cedar forest near stream, in leaf litter (2 ♂, NBM, RWC); Browns Mtn. Fen, 45.8967°N, 67.6343°W, 2.V.2005, M.-A. Giguère & R. Webster, eastern white cedar fen, in moist sphagnum in area with sedges (2 ♂, 2 ♀, NBM); New Maryland, off Hwy 2, E of Baker Brook, 45.8760°N, 66.6252°W, 28.IV.2005, 4.VI.2005, R. P. Webster, old-growth eastern white cedar swamp, in moss and litter at base of cedar (2 ♂, NBM); 9 km W of Tracy, off Rt.645, 45.6888°N, 66.8004°W, 22.V.2008, R. P. Webster, *Carex* marsh in hummock (1 ♀, NBM).

####### Collection and habitat data.

This species was mostly found in sphagnum bogs and swamps, and adults were collected by sifting sphagnumand other mosses, leaf litter, grasses, cedar duff or treading vegetation into water (Smetana 1995). In New Brunswick, most adults were found in eastern white cedar swamps or tamarack bogs and were collected either by treading or sifting moist sphagnum. Adults were collected in April, May, June, and September.

####### Distribution in Canada and Alaska.

AB, MB, ON, QC, **NB** (Smetana 1995).

**Map 47.**
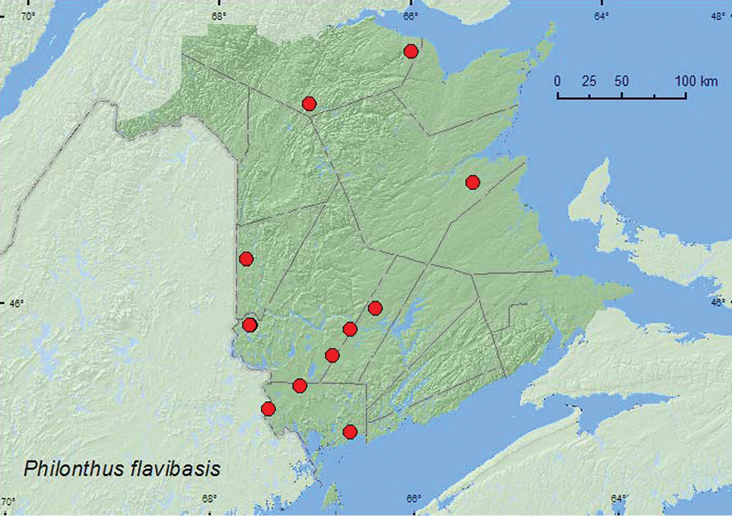
Collection localities in New Brunswick, Canada of *Philonthus flavibasis*.

###### Philonthus janus

Smetana, 1995**

http://species-id.net/wiki/Philonthus_janus

Map 48

####### Material examined.

**New Brunswick, York Co.**, Charters Settlement, 45.8267°N, 66.7343°W, 14.V.2005, 21.V.2006, R. P. Webster, *Carex* marsh / fen, treading *Carex* hummocks (1 ♂, 1 ♀, RWC).

####### Collection and habitat data.

This species occurs in wet habitats such as marshes and can be collected by treading vegetation into water. This species has also been collected in numbers from beaver lodges and muskrat nests (Smetana 1995). The two New Brunswick specimens were collected during May by treading *Carex* hummocks in a *Carex* marsh.

####### Distribution in Canada and Alaska.

SK, MB, ON, QC, **NB** (Smetana 1995).

**Map 48.**
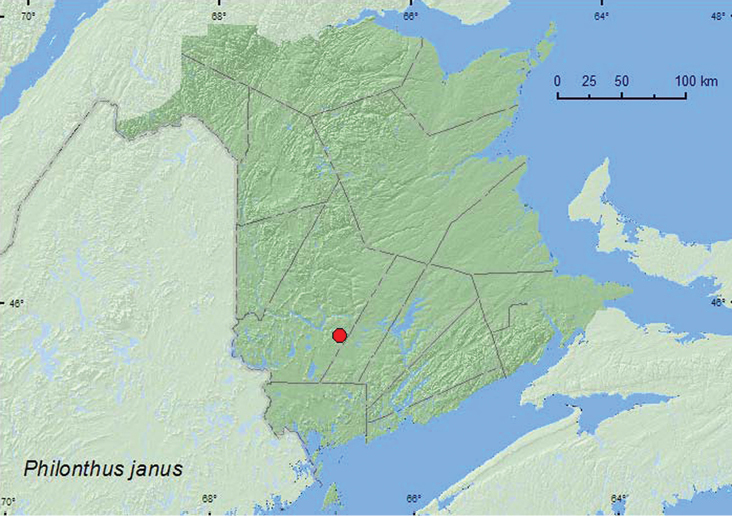
Collection localities in New Brunswick, Canada of *Philonthus janus*.

###### Philonthus monaeses

Smetana, 1995

http://species-id.net/wiki/Philonthus_monaeses

Map 49

####### Material examined.

**New Brunswick, Carleton Co.**, Jackson Falls, Bell Forest, 46.2150°N, 67.7201°W, 14.V.2006, R. P. Webster, river margin in drift material near seepage area (1 ♂, RWC). **Charlotte Co.**, near Clark Ridge, 45.3155°N, 67.4406°W, 27.V.2007, R. P. Webster, beaver pond, treading (floating) vegetation (1 ♀, RWC); 5.2 km NW of Pomeroy Ridge, 45.3087°N, 67.4362°W, 5.VI.2008, R. P. Webster, red maple swamp, in leaf litter near vernal pool (1 ♂, RWC). **Queens Co.**, Grand Lake near Indian Point, 45.8762°N, 66.1816°W, 5.VI.2004, R. P. Webster, lake margin under drift material (3 ♀, RWC). **Sunbury Co.**, Maugerville, Portobello Creek N.W.A., 45.8992°N, 66.4248°W, 27.V.2004, R. P. Webster, silver maple swamp, margin of vernal pond in moist leaf litter (1 ♀, RWC). **York Co.**, Dumfries, Slagundy Dry Ponds, 45.8596°N, 67.1849°W, 8.VII.2006, R. P. Webster, large vernal pond, in moist leaves near water (1 ♂, RWC); 8.5 km W of Tracy off Rt. 645, 45.6888°N, 66.8004°W, 22.V.2008, R. P. Webster, *Carex* marsh/flowage, near slow flowing brook in *Carex* hummocks (1 ♂, RWC).

####### Collection and habitat data.

This species occurs in a wide variety of wetland habitats, usually associated with standing water such as wet meadows, marshes, swamps, forest seepages, and along pond and lake margins (Smetana 1995). Some individuals were collected from muskrat nests. Adults were collected by sifting leaf litter, grass, and moss or by treading vegetation into water. In New Brunswick, this species was found in similar kinds of habitats. Adults were found in drift material near a seepage area along a river margin, among moist leaves on vernal pond margins in red maple and silver maple swamps, in a *Carex* hummock in a *Carex* flowage/marsh, in floating vegetation on a beaver pond margin, and in drift material on a lake margin. Adults were collected during May, June, and July.

####### Distribution in Canada and Alaska.

ON, QC, **NB**, (Smetana 1995).

**Map 49.**
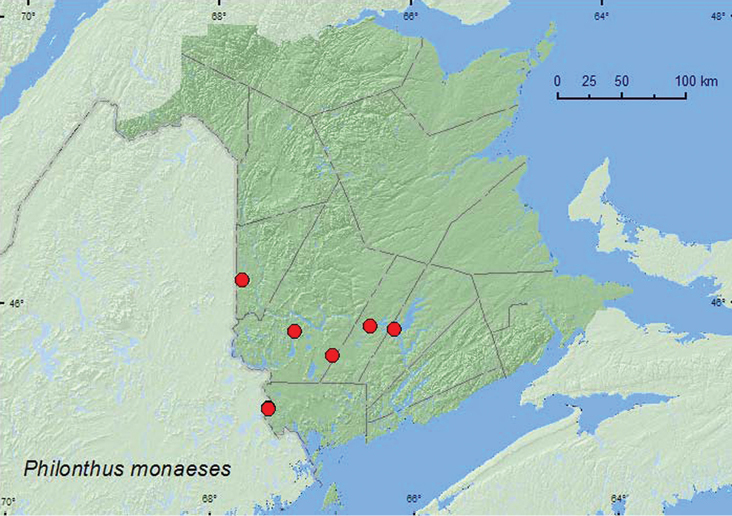
Collection localities in New Brunswick, Canada of *Philonthus monaeses*

###### Philonthus neonatus

Smetana, 1965

http://species-id.net/wiki/Philonthus_neonatus

Map 50

####### Materal examined.

**New Brunswick, Carleton Co.**, Meduxnekeag Valley Nature Preserve, 46.1931°N, 67.6825°W, 13.VII.2004, R. P. Webster, river margin, under drift material (1 ♀, RWC); Becaguimec Island, 46.3073°N, 67.5376°W, 23.VI.2006, R. Capozi & R. Webster, river margin, on sand near water (1 ♂, RWC). **Queens Co.**, Bayard at Nerepis River, 45.4473°N, 66.3318°W, 24.V.2009, R. P. Webster, river margin, on sand bar, in debris on sand (7 ♂, 1 ♀, RWC).

####### Collection and habitat data.

*Philonthus neonatus*is generally a riparian species occurring along the margins of creeks and smaller rivers and beaches of larger lakes, usually in flood debris (Smetana 1995). The New Brunswick specimens were collected from similar habitats during May and June. Adults were collected along river margins from under drift material, on sand and from debris resting on sand on a sand bar.

####### Distribution in Canada and Alaska. 

ON, QC, **NB**, NS (Smetana 1995).

**Map 50.**
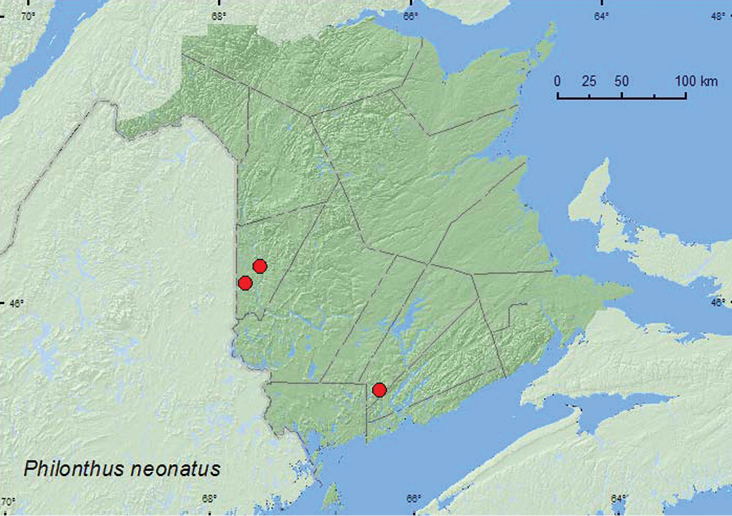
Collection localities in New Brunswick, Canada of *Philonthus neonatus*.

###### Philonthus pseudolus

Smetana, 1995**

http://species-id.net/wiki/Philonthus_pseudolus

Map 51

####### Material examined.

**New Brunswick, Carleton Co.**, Trail to “Two Mile Brook Fen”, 46.3600°N, 67.6815°W, 10.V.2005, R. P. Webster, cattail /*Carex* marsh, treading *Carex* hummocks into water (1 ♂, RWC). **Madawaska Co.**, Third Lake, 47.7786°N, 68.3783°W, 21.VI.2010, R. P. Webster, lake margin, in gravel among scattered sedges (1 ♂, RWC). **Queens Co.**, Grand Lake near Scotchtown, 45.8762°N, 66.1816°W, 30.IV.2006, R. P. Webster, lake shore, in drift material (1 ♂, RWC). **York Co.**, 8.5 km W of Tracy off Rt. 645, 45.6888°N, 66.8004°W, 22.V.2008, R. P. Webster, *Carex* marsh / flowage near slow flowing brook, in *Carex* hummock (1 ♂, RWC).

####### Collection and habitat data.

This species occurs in moist to wet habitats such as marshes, swamps, marshy margins of lakes and ponds and in muskrat nests. Adults are collected by treading vegetation (*Carex* and *Typha* and other vegetation) into water (Smetana 1995). In New Brunswick, adults were collected by treading *Carex* hummocks in *Carex* marshes and sifting drift material along lake margins. Adults were collected during April, May, and June.

####### Distribution in Canada and Alaska.

BC, AB, MB, ON, QC, **NB** (Smetana 1995).

**Map 51.**
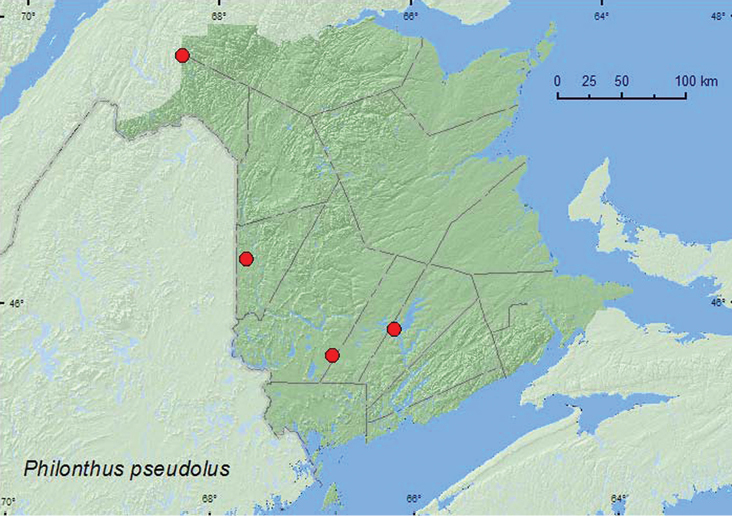
Collection localities in New Brunswick, Canada of *Philonthus pseudolus*.

###### Philonthus sericinus

Horn, 1884**

http://species-id.net/wiki/Philonthus_sericinus

Map 52

####### Material examined.

**New Brunswick, Carleton Co.**, Meduxnekeag River Valley Nature Preserve, 46.1907°N, 67.6740°W, 14.IX.2005, R. P. Webster, mixed forest, in decaying mushrooms (1 ♂, RWC). **Sunbury Co.**, Acadia Research Forest, 46.0173°N, 66.3741°W, 17–23.VIII.2007, R. P. Webster, 8.5-year-old regenerating mixed forest, pitfall trap (1, AFC). **York Co.**, Charters Settlement, 45.8395°N, 66.7391°W, 17.VII.2004, 6.V.2008, R. P. Webster, mixed forest, in compost (decaying vegetables) (2 ♂, RWC); same locality and collector, 45.8430°N, 66.7275°W, 17.IX.2004, 25.IX.2004, regenerating mixed forest, baited with pile of decaying mushrooms (1 ♂, 4 ♀, RWC); same locality data and collector, 5.VI.2004, under carrion (1 ♀, RWC).

####### Collection and habitat data.

This species occurs in various rotting organic materials such as compost, rotting grass clippings, decaying fruit, vegetables, and decaying mushrooms, and rarely in forest floor leaf litter (Smetana 1995). In New Brunswick, most adults were found in decaying mushrooms in mixed forests. Adults were collected in May, June, July, August, and September.

####### Distribution in Canada and Alaska.

ON, QC, **NB** (Smetana 1995).

**Map 52.**
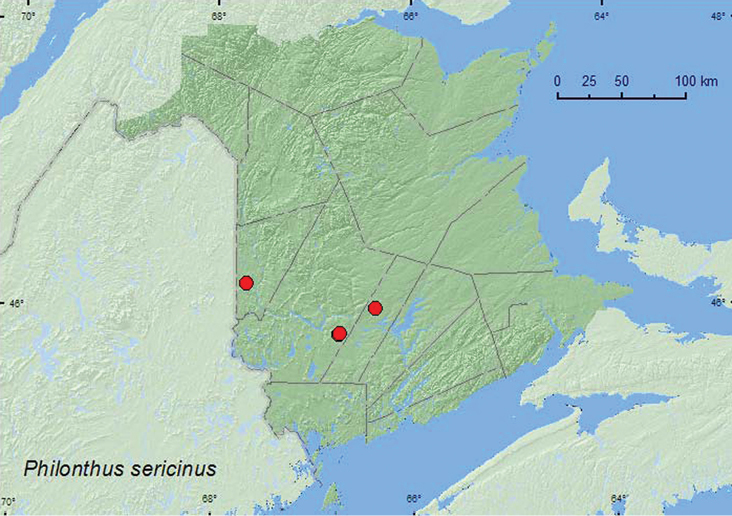
Collection localities in New Brunswick, Canada of *Philonthus sericinus*.

###### Philonthus subvirescens

Thomson, 1884**

http://species-id.net/wiki/Philonthus_subvirescens

Map 53

####### Material examined.

**New Brunswick, Madawaska Co.**, at Green River, 47.6918°N, 68.3202°W, 21.VI.2010, R. P. Webster, river margin among gravel on gravel bar (1 ♂, RWC). **Restigouche Co.**, Kedgwick Forks, 47.9085°N, 67.9057°W, 22.VI.2010, R. P. Webster, river margin, in flood debris, on gravel bar among gravel and cobblestones (4 ♂, RWC).

####### Collection and habitat data.

*Philonthus subvirescens* is a riparian species found on sandy and gravel margins in areas with sparse vegetation (Smetana 1995). This species was found among gravel and flood debris along the margins of small fast-flowing rivers in New Brunswick.

####### Distribution in Canada and Alaska.

AK, YT, NT, BC, AB, QC, **NB** (Smetana 1995). *Philonthus subvirescens* is a Holarctic species with a mostly western distribution from Alaska and the Northwest Territories south to the mountains of California and east to New Mexico, with a significantly disjunct population in Quebec on the Gaspé Peninsula (Smetana 1995). The presence of this species in northwestern New Brunswick indicates that this species has a somewhat wider distribution in easternmost Canada.

**Map 53.**
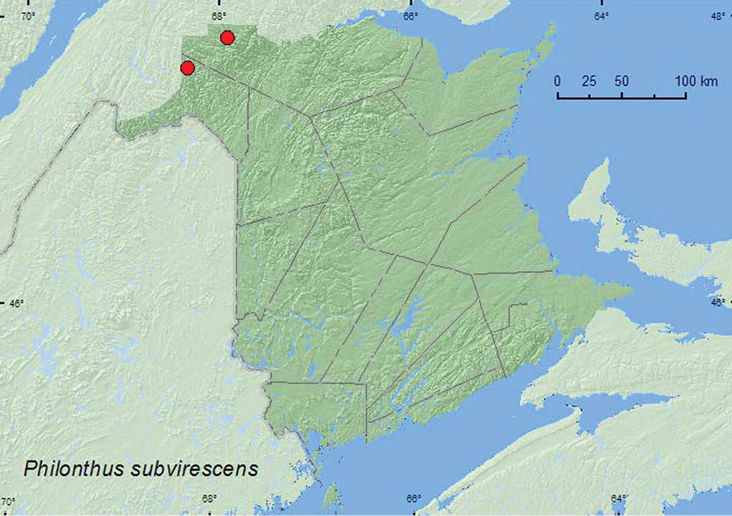
Collection localities in New Brunswick, Canada of *Philonthus subvirescens*.

###### Philonthus thoracicus

(Gravenhorst, 1802)

http://species-id.net/wiki/Philonthus_thoracicus

Map 54

####### Material examined.

**Additional New Brunswick records. Carleton Co.**, Becaguimec Island, 46.3106°N, 67.5392°W, 16.IX.2006, R. Capozi & R. Webster, hardwood forest (on island in Saint John River), on *Pleurotus* sp. on log (1 ♂, RWC). **Queens Co.**, W of Jemseg at “Trout Creek”, 45.8231°N, 66.1245°W, 3.IV.2006, R. P. Webster, silver maple swamp, sifting litter from crotch silver maple with multiple trunks (1 ♀, RWC). **Sunbury Co.**, Lakeville Corner, 45.9008°N, 66.2414°W, 12.VII.2006, R. P. Webster, silver maple swamp on ridge with red oak and red maple, in litter at base of tree (1 ♀, RWC). **York Co.** Fredericton, Nashwaaksis River at Rt. 105, 45.9850°N, 66.6900°W, R. P. Webster, in flood debris on upper river margin (1 ♂, 1 ♀, RWC).

####### Collection and habitat data.

This species has been found in open, often dry habitats with sandy substrates (open pine forests) and in moist to wet habitats near water, such as sandy creek, river, and pond margins (Smetana 1995). Adults occurred in leaf litter and debris. Specimens were also found in entrances of *Marmota* burrows (Smetana 1995). In New Brunswick, this species was found in silver maple floodplain forests, a hardwood forest on an island in a large river, and on an upper river margin. Adults were collected from leaf litter from a crotch of a silver maple with multiple trunks, litter at the base of a tree, in flood debris, and from *Pleurotus* mushrooms on a log.

####### Distribution in Canada and Alaska.

AB, SK, MB, ON, QC, NB (Smetana 1995). *Philonthus thoracicus* was previously known from New Brunswick from one specimen collected by G.A. Calderwood in the Kouchibouguac National Park (Smetana 1995). It is apparent from the above records that this species is more widely distributed in the Maritime provinces than was suggested by the distributional gaps shown in Majka et al. (2009). Floodplain forests should be sampled for this species in Nova Scotia.

**Map 54.**
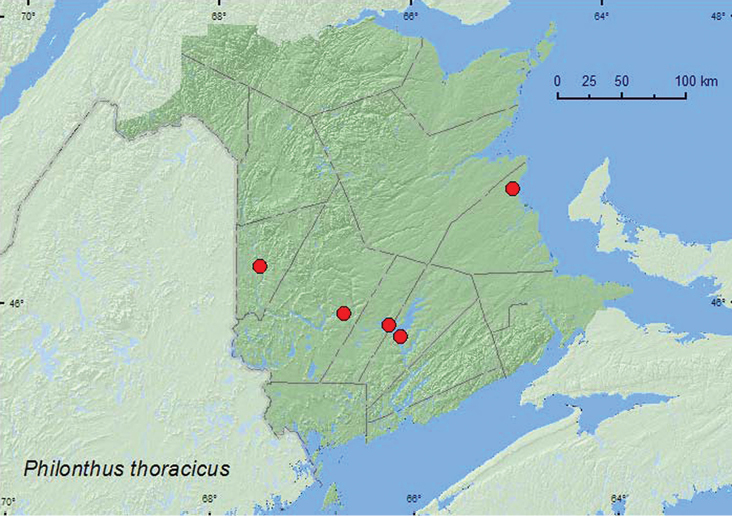
Collection localities in New Brunswick, Canada of *Philonthus thoracicus*.

###### Philonthus umbrinoides

Smetana, 1995

http://species-id.net/wiki/Philonthus_umbrinoides

Map 55

####### Material examined.

**New Brunswick, Carleton Co.**, “Two Mile Brook Fen”, 46.3594°N, 67.6800°W, 2.VI.2005, R. P. Webster, on (dirt) road through eastern white cedar swamp, in flight late afternoon (1 ♀, RWC). **Charlotte Co.**, near Clark Ridge, 45.3155°N, 67.4406°W, 26.V.2007, R. P. Webster, beaver pond, treading vegetation (1 ♀, RWC). **Queens Co.**, Grand Lake near Scotchtown, 45.8762°N, 66.1816°W, 12.V.2004, R. P. Webster, lakeshore, under drift material (1 ♂, RWC). **Sunbury Co.**, Sheffield, Portobello Creek N.W.A., 45.8952°N, 66.2728°W, 7.V.2004, R. P. Webster, silver maple swamp, in leaf litter (1 ♂, RWC).

####### Collection and habitat data.

This species occurs in moist to wet habitats such as marshes, swamps, marshy margins of lakes and ponds, and in muskrat nests. Adults have been collected by treading vegetation (floating sphagnum mats, sedges and mosses, *Typha* plants, reeds) into water (Smetana 1995). In New Brunswick, adults were collected by treading vegetation along a beaver pond margin, from under drift material on a lake margin, sifting leaf litter in a silver maple swamp, and in flight during evening. Adults were collected in May and June.

####### Distribution in Canada and Alaska.

AB, MB, ON, QC, **NB**, NS (Smetana 1995).

**Map 56.**
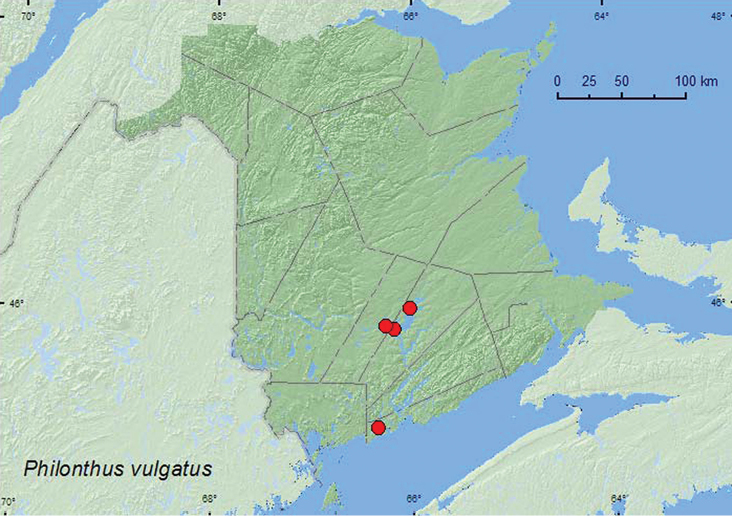
Collection localities in New Brunswick, Canada of *Philonthus vulgatus*.

###### Philonthus vulgatus

Casey, 1915

http://species-id.net/wiki/Philonthus_vulgatus

Map 56

####### Material examined.

**New Brunswick, Queens Co.**, Grand Lake near Scotchtown, 45.8762°N, 66.1816°W, 5.VI.2004, R. P. Webster, lakeshore, under drift material (1 ♀, RWC); same locality and collector, 9.VII.2006, oak maple forest near lakeshore, m.v. light (1 ♂, 3 ♀, RWC); Grand Lake at Flowers Cove, 46.0196°N, 66.0246°W, 26.VIII.2004, D. Sabine & R. Webster, lake margin, under drift material (1 ♀, RWC). **Saint John Co.**, Musquash, 45.1856°N, 66.3402°W, 30.V.2006, R. P. Webster, *Carex* and cattail marsh, treading (1 ♂, RWC). **Sunbury Co.**, Sheffield, Portobello Creek N.W.A., 45.8952°N, 66.2728°W, R. P. Webster, 18.VI.2004, silver maple swamp, u.v. light trap (2 ♂, RWC).

####### Collection and habitat data.

*Philonthus vulgatus*occurs in debris along margins of ponds, lakes, swamps, marshes, creeks, and rivers, in beaver lodges and muskrat nests, and it commonly comes to light (Smetana 1995). In New Brunswick, adults were found under drift material along lake margins and at an ultraviolet light. Adults were collected during June, July, and August.

####### Distribution in Canada and Alaska.

AK, BC, AB, SK, MB, ON, QC, **NB**, NS, PE, NF (Smetana 1995; Brunke and Marshall 2011).

**Map 55.**
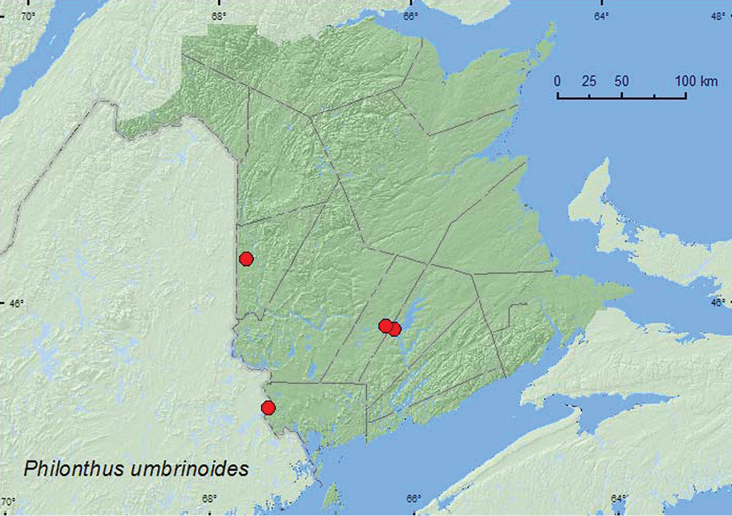
Collection localities in New Brunswick, Canada of *Philonthus umbrinoides*.

## Supplementary Material

XML Treatment for Diochus schaumi

XML Treatment for Atrecus americanus

XML Treatment for Gyrohypnus campbelli

XML Treatment for Hypnogyra gularis

XML Treatment for Neohypnus beckeri

XML Treatment for Phacophallus parumpunctatus

XML Treatment for Xestolinus abdominalis

XML Treatment for Acylophorus (Amacylophorus) pratensis

XML Treatment for Acylophorus (Amacylophorus) caseyi

XML Treatment for Hemiquedius ferox

XML Treatment for Heterothops minor

XML Treatment for Heterothops pusio

XML Treatment for Quedius (Microsaurus) campbelli

XML Treatment for Quedius (Microsaurus) canadensis

XML Treatment for Quedius (Microsaurus) criddlei

XML Treatment for Quedius (Microsaurus) erythrogaster

XML Treatment for Quedius (Microsaurus) mesomelinus

XML Treatment for Quedius (Microsaurus) peregrinus

XML Treatment for Quedius (Quedius) curtipennis

XML Treatment for Quedius (Quedius) labradorensis labradorensis

XML Treatment for Quedius (Quedionuchus) plagiatus

XML Treatment for Quedius (Distichalius) capucinus

XML Treatment for Quedius (Distichalius) cinctus

XML Treatment for Quedius (Raphirus) frigidus

XML Treatment for Quedius (Raphirus) fulvicollis

XML Treatment for Quedius (Raphirus) simulator

XML Treatment for Staphylinus ornaticauda

XML Treatment for Bisnius cephalicus

XML Treatment for Bisnius cephalotes

XML Treatment for Bisnius palmi

XML Treatment for Bisnius quediinus

XML Treatment for Erichsonius alumnus

XML Treatment for Erichsonius inutilis

XML Treatment for Erichsonius parcus

XML Treatment for Erichsonius patella

XML Treatment for Erichsonius pusio

XML Treatment for Erichsonius rosellus

XML Treatment for Gabrius appendiculatus

XML Treatment for Gabrius fallaciosus

XML Treatment for Gabrius ulpius

XML Treatment for Hesperus apicialis

XML Treatment for Laetulonthus laetulus

XML Treatment for Neobisnius jucundus

XML Treatment for Neobisnius lathrobioides

XML Treatment for Neobisnius terminalis

XML Treatment for Philonthus aequalis

XML Treatment for Philonthus boreas

XML Treatment for Philonthus flavibasis

XML Treatment for Philonthus janus

XML Treatment for Philonthus monaeses

XML Treatment for Philonthus neonatus

XML Treatment for Philonthus pseudolus

XML Treatment for Philonthus sericinus

XML Treatment for Philonthus subvirescens

XML Treatment for Philonthus thoracicus

XML Treatment for Philonthus umbrinoides

XML Treatment for Philonthus vulgatus
